# Preparation and Characterization of Chitosan-Alginate Microspheres Loaded with Quercetin

**DOI:** 10.3390/polym14030490

**Published:** 2022-01-26

**Authors:** Olimpia Daniela Frenț, Narcis Duteanu, Alin Cristian Teusdea, Stefania Ciocan, Laura Vicaș, Tunde Jurca, Mariana Muresan, Annamaria Pallag, Paula Ianasi, Eleonora Marian

**Affiliations:** 1 Doctoral School of Biomedical Science, University of Oradea, 1 University Street, 410087 Oradea, Romania; daniela.olimpia@yahoo.com; 2 Faculty of Industrial Chemistry and Environmental Engineering, Politehnica University of Timisoara, 2 Piata Victoriei, 300006 Timisoara, Romania; 3 Faculty of Environmental Protection, University of Oradea, 26th Gen. Magheru Avenue, 410087 Oradea, Romania; 4 Department of Pharmacy, Faculty of Medicine and Pharmacy, University of Oradea, 29 Nicolae Jiga Street, 410028 Oradea, Romania; jurcatunde@yahoo.com (T.J.); annamariapallag@gmail.com (A.P.); marian_eleonora@yahoo.com (E.M.); 5 Department of Preclinical Discipline, Faculty of Medicine and Pharmacy, University of Oradea, 10, 1th December Square, 410068 Oradea, Romania; marianamur2002@yahoo.com; 6 INCEMC-National Institute for Research and Development in Electrochemistry and Condensed Matter—Timisoara, No.144 Dr. A. Paunescu Podeanu Street, 300569 Timisoara, Romania; paulasvera@gmail.com

**Keywords:** microspheres, biodegradable polymers, complex coacervation, chitosan, sodium alginate, quercetin

## Abstract

The aim of this paper was to formulate microspheres based on biodegradable polymers (chitosan and sodium alginate), using the complex coacervation technique. Subsequently, the prepared microspheres were loaded with quercetin (QUE), a pharmacological active ingredient insoluble in water and unstable to light, temperature and air. After preparation, the loaded microspheres underwent several studies for physical chemical characterization (performed by scanning electron microscopy—SEM, laser 3D scanning, and thermal analysis—TA). Furthermore, they were analyzed in order to obtain information regarding swelling index, drug entrapment, and in vitro release capacity. The obtained experimental data demonstrated 86.07% entrapment of QUE into the microspheres, in the case of the one with the highest Ch concentration. Additionally, it was proved that such systems allow the controlled release of the active drug over 24 h at the intestinal level. SEM micrographs proved that the prepared microspheres have a wrinkled surface, with compact structures and a large number of folds. On the basis of the TA analysis, it was concluded that the obtained microspheres were thermally stable, facilitating their usage at normal physiological temperatures as drug delivery systems.

## 1. Introduction

At present, microencapsulated pharmaceutical forms can be used as particulate systems able to incorporate various natural or synthetic pharmacologically active substances. In this regard, for microsphere preparation, natural materials are used because they are biocompatible, biodegradable, non-carcinogenic, non-toxic, harmless and safe for the human body, and economical, while also being available, solving many of the problems related to the release and transport of pharmaceutical substances [1,2,3,4,5,6,7,8]. Nano-scale applications of organic compounds in the medical field have demonstrated that active drugs can be delivered using different nanocarriers [9,10,11,12]. Experiments carried out by Barani et al. proved that niosomes represent a viable compound for use as a drug delivery system [9].

Natural polymers include polysaccharide macromolecules of vegetable or animal origin, whose structure can be chemically modified, and is biocompatible with the body, without side effects [5,13]. Two natural biodegradable polymers, Ch and Na-Alg, were included in the microsphere matrix in this study. 

Ch, due to the cationic characteristic offered by the primary amino groups, has mucoadhesion, intensifying the penetration of the active substance through the intestinal mucosa, and facilitating the opening of the junctions of the intestinal epithelium [14], making paracellular and transcellular transport possible [15,16], while offering a high capacity for controlled release of the active drug substance; additionally, it can gel in situ, is non-toxic, non-allergenic, and is safe for the human body [17,18,19,20,21,22]. Ch itself, despite its many advantages as a polymer, is not suitable for use alone in the development of controlled-release pharmaceutical systems, because it disintegrates rapidly in gastric juice due to the acidic pH; therefore, it needs to be used in combination with various anionic polymers so that the resulting preparation is safe, stable, and presents efficient drug release in acidic environments [23]. The mechanism formation of Ch microparticles by complexation with Na-Alg is based on an electrostatic bond that occurs between the negative anionic groups of polyelectrolytes (Alg) and the cationic positive groups (amine groups) of Ch. The size of the complexes formed can vary between 50 and 700 µm [24,25,26]. 

Alg is also a natural, biodegradable, biocompatible polysaccharide that is safe for the human body, and it offers multiple advantages in the pharmaceutical industry: it ensures reversible and stable gels, it is cheap and available, it is able to quickly absorb water, resulting in the formation of a kind of viscous gums, and it can be used as a microencapsulation material to obtain microspheres that ensure the controlled release of drug substances from the pharmaceutical system [21,25,27,28].

QUE it is a flavonoid with polyphenolic structure with various therapeutic properties: antioxidant, antibacterial, antiviral, anticancer, anti-inflammatory, etc. In this study, QUE was included in the microspheres as an active drug substance. However, the use of QUE in the field of pharmaceuticals is limited due to its stability problems with respect to light, temperature, and oxygen exposure. The aim of the present study was to limit the stability problems of QUE by developing microencapsulated pharmaceuticals obtained by complex coacervation, using non-toxic, biodegradable and biocompatible microencapsulation materials (Ch and Na-Alg). These microencapsulated products are stable at high temperatures, and are effective at presenting controlled release [22,29,30,31,32]. 

## 2. Materials and Methods

### 2.1. Materials

Chitosan (with degree of deacetylation > 75%, and a molecular weight between 310,000 and 375,000 Da) and quercetin were purchased from Sigma Aldrich Co (Burlington, MA, US). Sodium alginate, sodium hydroxide 1 M, hydrochloric acid and calcium chloride were purchased from Chempur (Piekary Slaskie, Poland). Acetic acid, sodium acetate, potassium dihydrogen phosphate, sodium dihydrogen phosphate, disodium hydrogen phosphate and sodium citrate were purchased from Promochem (Wesel, Germany). All reagents were used as such in the research showed pharmaceutical or analytical purity.

Equipment: analytical balance type Kern ABT 220-5DNM (Kern and Sohn GmbH, Balingen, Germany), ultrasonic bath type Elmasonic S 100 H (Elma Schmidbauer GmbH, Singen, Germany), pH meter type pH 7310 Inolab (Xylem Analytics GmbH, Wellheim, Germany), Ecostris magnetic stirrer (Dlab Scirntific Co., Ltd., Beijing, China), Universal 320R centrifuge (Hettich GmbH and Co KG, Tuttingen, Germany), FTIR 7800 (PG Instruments Ltd., Lutterworth, Great Britain), UV-VIS spectrophotometer T70+ (PG Instruments Ltd., Lutterworth, Great Britain), Ratiopetta pipette 1000-5000 µL (Ratiolab GmbH, Dreieich, Germany), 20 mL Becton Dickinson Discarded syringe with 23G needle (0.60 mm× 30 mm) KDM.

### 2.2. Methods

#### Preparation of Microspheres CH-Alg-QUE

For preparation of microspheres, Na-Alg (0.75% (*v*/*v*)) and two Ch (0.1% and 0.2% (*v*/*v*)) stock solutions were used. Ch solutions were prepared by dissolution of the required amount of Ch (0.1 or 0.2 g) in 100 mL of freshly prepared acetic acid solution (with a concentration of 2%). After the Ch was added into the acetic acid solution, the system was kept in contact for about 20 min to allow Ch hydration, followed by a mixing process using a magnetic stirrer at 100 rotations per minute for 30 min. Furthermore, in order to obtain proper homogenization of the prepared Ch solutions, recipients were ultrasonicated for 30 min. Finally, 4 g of calcium chloride was added, and the pH was adjusted to 5.5 by adding needed quantity of 10% sodium hydroxide solution. Na-Alg solution was prepared by dispersing 0.75 g Na-Alg in 99.25 g of distilled water, and contact was maintained for a minimum of 30 min in order to allow Na-Alg hydration. Obtained solution was stirred magnetically at 100 rpm for minimum 30 min in order to prevent Na-Alg agglomeration and to improve its dissolution. Furthermore, air bubbles formed during dissolution were eliminated by solution ultrasonication for minimum 20 min. For microsphere preparation, 50 mL Ch stock solution and 20 mL Na-Alg solution were used. The obtained composition of the prepared microspheres is presented in Table 1. For subsequent incorporation of QUE into the prepared microspheres, the required quantity of pure QUE was dissolved into the freshly prepared Na-Alg solution.

**Figure 1:**
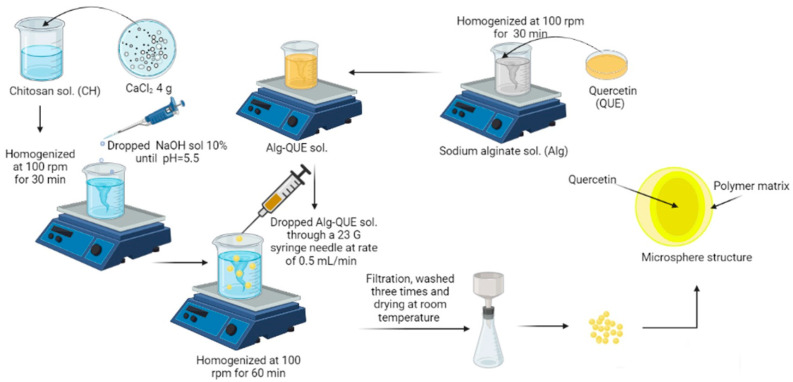
Preparation of quercetin microspheres by complex coacervation.

A schematic diagram of the microsphere preparation is presented in Figure 1. After preparation of stock solutions, first, the right amount of Na-Alg-QUE was taken using a sterile hypodermic syringe, needle size 23 G. Then, Na-Alg/QUE solution was added drop by drop, at a constant rate of 0.3 mL min^−1^, into the Ch solution. The obtained microspheres were separated by filtration, and were washed three times with distilled water and once with acetone. The cleaned microspheres were dried in the oven for 24 h at 30 °C until constant mass. Dried microspheres were kept in hermetically sealed containers at room temperature (25 °C) until further analysis. A similar procedure was used for further preparation of microspheres without QUE content (P4 and P8).

With respect to which kinds of interactions dominate the encapsulation process, a possible mechanism has been proposed that tries to explain the microsphere formulation. This mechanism was proposed by extrapolation of the data presented in the paper published by Lopez-Maldonado et al. [22], and is depicted in Figure S4 in the Supplementary Materials. Based on this, it can be concluded that the microsphere formation and the drug loading are the consequence of the electrostatic forces exhibited between the positive and negative charged groups of Ch, Na-Alg, and QUE.

## 3. Characterization of Microspheres

### 3.1. Determination of Quercetin Entrapment Efficiency (EE (%)) into the CH-Alg Microspheres

Efficiency of QUE entrapment in the prepared microspheres was determined by recording specific UV-VIS spectra. The first step was to determine the calibration plot obtained from spectra recorded when different quantities of pure QUE were dissolved into the 5% sodium citrate solution. 

To record such specific spectra, a 0.025 g quantity of microspheres was introduced into 100 mL of 5% sodium citrate solution, and magnetically stirred for 6 h at 500 rpm. After that, for proper separation, the obtained mixture was centrifuged at 1500 rpm at 37 °C for 15 min. UV-VIS spectra were recorded by taking a sample from the supernatant, and reading the absorbance at 376 nm (specific for QUE molecules) [33]. 

Entrapment efficiency was calculated using Equation (1):

(1) $$EE\%=\frac{C_{pQue\;e}}{C_{tQue\;e}}\times 100\;\;$$

where:

$C_{pQue\;e}$ = practical concentration of QUE encapsulated;

$C_{tQue\;e}$ = theoretical concentration of QUE encapsulated. 

The experiment was performed in triplicate and the results are expressed as mean ± SD (standard deviation).

### 3.2. Swelling Index Study

To determine the swelling index of the prepared microspheres, five dry microspheres, with a well-known mass, were suspended in 3 mL acetate buffer solutions with different pH values (1.2, 3.0, 5.0) and phosphate buffer solutions with different pH values (6.8 and 7.4). All these solutions were prepared in accordance with the Romanian Pharmacopoeia Xth edition and the previous work performed by Ranjha N.M and Qureshi U.F. in 2014 [34,35]. Furthermore, the swelling index of the microspheres was calculated using Equation (2). For this, swollen microspheres were weighed after excess water had been removed by wiping with Whatman filter paper. This analysis was performed for 8 h, and the weighing was performed using a Kern ABT 220-% DNM analytical balance (Kern and Sohn GmbH, Balingen, Germany) until sample mass reached a constant weight. All the experiments were carried out in triplicate and the obtained results are presented as mean ± SD.

(2) $$\mathrm{Swelling}\;\mathrm{index}\;\left(\%\right)=\frac{M_{sm}-M_{dm}}{M_{dm}}\times 100$$

where $M_{sm}$—mass of swollen microspheres, $M_{dm}$—mass of dry microspheres. 

### 3.3. In Vitro Release Studies of Quercetin Microspheres

The release tests for QUE contained in the prepared microspheres were performed using the official method described in the United States Pharmacopoeia 2020 (USP 43) using an Electrolab TDT-08L-Dissolution tester [13]. Based on the recommendation in USP 43, the working conditions for the release tests were as follows: constant temperature of 37 °C, dissolution time—24 h, rotation speed of the device baskets—30 rpm, dissolution medium—phosphate buffer solution with pH of 7.4 in order to simulate real intestinal juice medium. Phosphate buffer medium was prepared in accordance with the Romanian Pharmacopoeia Xth edition [35], and for each basket, a quantity of 400 mL was used. The times at which samples were collected from each basket were as follows: 0.25, 0.5, 0.75, 1, 2, 3, 4, 5, 6, 12 and 24 h. After sample collection, each one was filtered using Whatman filter paper, and the QUE content was then determined by measuring the absorbance at 376 nm by UV-VIS spectroscopy. All the experiments were carried out in triplicate and the obtained results are presented as mean ± SD.

### 3.4. Thermal Analysis

Thermogravimetric analysis was performed in order to determine the thermal stability of the prepared microspheres. All these analyses were performed using a Netzsch TG 29 F1 Libra system (Netzsch Geratebau GmbH, Selb, Germany). The samples were heated within the temperature range between 25 and 950 °C, at a constant rate of 10 k min^−1^, under a nitrogen atmosphere. Gas flow was 40 mL min^−1^, and the use of nitrogen was chosen in order to ensure an inert and protective atmosphere during measurements. 

### 3.5. Scanning Laser Confocal Microscopy Analysis

Microsphere roughness was determined by performing 3D measurements using a laser 3D measuring microscope (Lext OLS 4000, Olympus, Tokyo, Japan). In this case, all 2D images were recorded, followed by 3D measurement using a 405-nm laser light source. From these images, an image profile was obtained, and the roughness data were further calculated. 

### 3.6. Scanning Electron Microscopy 

The obtained microspheres were characterized by scanning electron microscopy, SEM, using a FEI Quanta FEG 250 microscope (FEI, Hillsboro, OR, USA). For each sample, five microspheres were used, which were placed onto the microscope holder. Due to the organic nature of the samples, SEM pictures were recorded using low vacuum and low voltage [36]. These conditions were needed in order to do not affect the analyzed samples.

### 3.7. Statistical Analysis

In the DOE (Design of Experiment), the following parameters were used with one statistical factor: entrapment efficiency (EE (%)) and roughness data (Sa and Sq (µm)), and means of these parameters were compared using one-way ANOVA (Analysis of Variance, *p* = 0.05). 

The means of the swelling index (idxSWL(%)) were compared with three-way ANOVA (Analysis of Variance, *p* = 0.05) for the following factors: 

Sample with levels: P1, P2, P3, P5, P6, P7 and QUE (see Table 1).

pH with levels: 1.2, 3, 5, 6.8 and 7.4.

Time t(h) with levels: 0.25, 0.5, 0.75, 1, 2, 3, 4, 5, 6, 8, 12 and 24 h. 

The means of quercetin in vitro release (mQUErel(%)) were compared using a two-way ANOVA (Analysis of Variance, *p* = 0.05), only for pH = 7.4, for the following factors: 

Sample with levels: P1, P2, P3, P5, P6, P7 and QUE (see Table 1).

Time t(h) with levels: 0.25, 0.5, 0.75, 1, 2, 3, 4, 5, 6, 8, 12 and 24 h.

A multivariate statistical sequence was used, consisting of: PCA (Principal Component Analysis), MANOVA (Multivariate ANOVA *p* = 0.05), and AHC (Agglomerative Hierarchical Cluster Analysis). The multivariate analysis was performed in order to decide which sample had the best combined performance for quercetin release and the best microsphere surfactant roughness.

All sample parametric data were analyzed in triplicate and the obtained results are presented as mean ± SD. The statistical analysis and graphical representations were performed with homemade subroutines including standardized statistical methods in MATLAB software (MatWorks Inc., 1 Apple Hill Drive, Natick, MA, USA). 

## 4. Results and Discussion

### 4.1. QUE Entrapment Efficiency (EE (%)) Study Results

QUE entrapment efficiency (EE (%)) was determined using UV-VIS spectrophotometry. In this context, firstly, the calibration curve for QUE was recorded. The linear form of the calibration curve is expressed by the equation: y = 5.4857 × X + 0.0381, where y = solution absorbance recorded at 376 nm expressed as absorbance units and x = QUE concentration expressed as mmol L^−1^.

On the basis of the specific absorbance obtained, the EE (%) was determined for each sample, and the results are presented in Table 2.

Analyzing the data presented in Table 2, it can be observed that EE (%) depends on the Ch concentration in the solutions used for microsphere preparation. As a consequence of increased Ch concentration, the quantity of QUE entrapped in the prepared microspheres can be observed to increase. 

**Figure 2:**
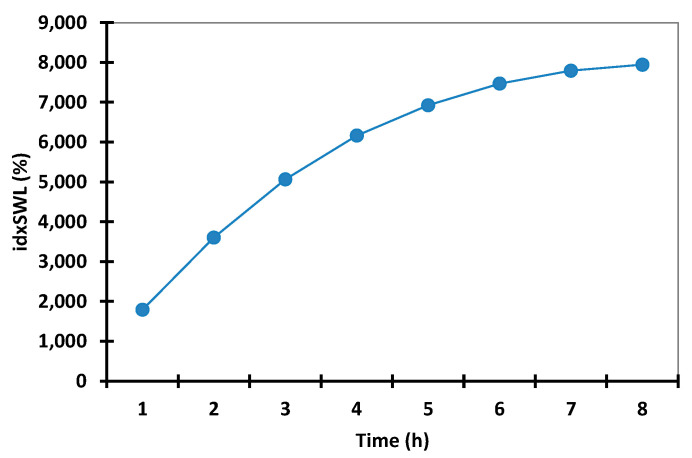
Interval plots of the microspheres’ swelling index (idxSWL (%)) from microspheres for Time factor levels.

The lowest QUE quantity entrapped (~82%) was obtained in the case of sample P1 (with a ratio Ch:Na-Alg: QUE = 0.1:0.75:0.05), while the highest quantity (~85%) was obtained in the case of sample P5 (with a ratio Ch:Na-Alg: QUE = 0.2:0.75:0.05), as reported in Figure 2. For samples P2 and P3, the quantity of entrapped QUE varied between the quantities obtained for samples P1 and P5. 

Likewise, samples P6 (0.2:0.75:0.075) and P7 (0.2:0.75:0.1) present the highest EE (%) values (85% and 86%, respectively). The P6 and P7 microspheres contained QUE amounts comparable to the amounts described by Scarfato et al., who prepared the microspheres via a solvent evaporation method using cellulose acetate phthalate or cellulose acetate propionate. Other methods used for microsphere preparation include an oil-in-water emulsion process, following the standard procedure with poly (lactic-*co*-glycolic acid) [37]. 

### 4.2. Swelling Index (idxSWL (%)) Study Results 

In preliminary tests (data are presented in Table S1 in the Supplementary Materials), swelling time was gradually increased in order to determine the optimal time at which the microspheres begin to degrade. On the basis of this experiment (data presented in Figure 2), it was observed that after 8 h, the microspheres reached their maximum swelling index threshold, and after 12 h, the microspheres started to break apart, which is in accordance with the results obtained from in vitro studies. 

**Figure 3:**
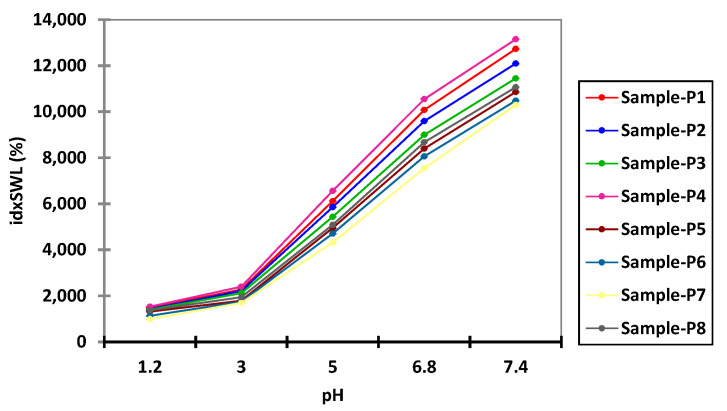
Interval plots of the swelling index (idxSWL (%)) of the microspheres for Sample * pH interaction factor levels.

**Figure 4:**
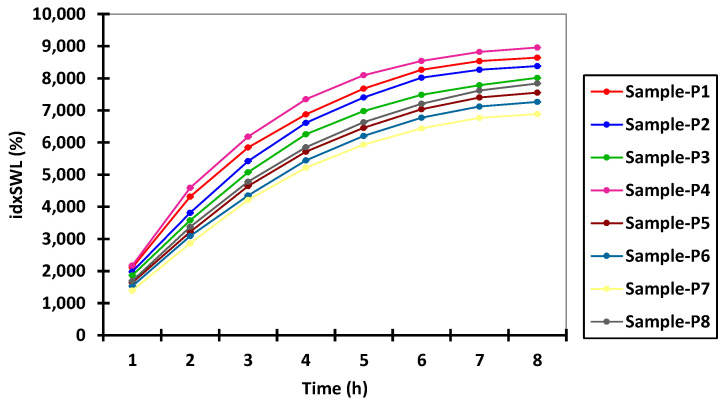
Interval plots of the swelling index (idxSWL (%)) of the microspheres for Sample * Time interaction factor levels.

In this context, according to the data presented in Figure 2, any further swelling experiments were carried out over a period of 8 h in both acidic and alkaline pH environments. Tables S2 and S3, in the Supplementary Materials, present the experimental data obtained from the water capture experiment, which was carried out using different time intervals and with different pH values. Figure 3 and Figure 4 present the experimental data obtained from the water capture experiment, which was carried out using different time intervals and with different pH values.

By analyzing the data obtained from the swelling experiments, it can be observed that the swelling process of the microspheres was a rapid one in environments with a pH of 6.8 and 7.4, and a slow one in environments with a pH of 1.2, 3 and 5, because it is influenced by the concentration of both Ch and QUE. Additionally, it can be observed from the data presented in Table 3 that, for all eight samples, the highest values for swelling index were obtained when the experiments were carried out at pH 6.8 (8.984%) and pH 7.4 (11.514%).

On the basis of the presented in Table 3, it can be concluded that the lowest swelling index was obtained at pH 1.2, whereby the samples remained intact after 8 h of analysis. Such behavior can be associated with the cation character of the Ch molecules provided by the presence of the amino groups in its structure. Such groups are ionizable, as they are positively charged at lower pH, and are complexed by the negative anionic groups found in the Na-Alg molecules. In this way, the improved stability of the prepared microspheres when exposed to a solution with acidic pH (1.2, 3.0, and 5.0) can be explained. When exposing the prepared microspheres to solutions with values of pH that were near neutral (6.8), or slightly basic (7.4), amino groups from Ch molecules presented a low charge density, which led to a decomplexation of Ch-Na-Alg complex, causing high water adsorption followed by rupture of the complex [38]. 

**Figure 5:**
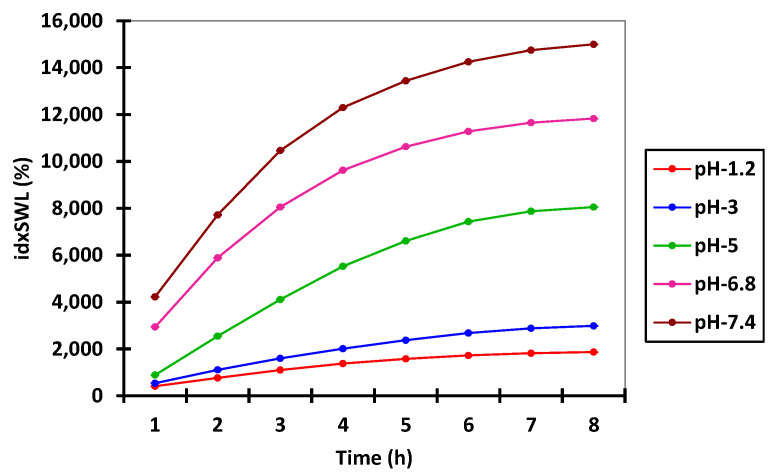
Interval plots of the swelling index (idxSWL (%)) of microspheres for pH * Time interaction factor levels.

On the basis of the data presented in Figure 5, it can be observed that for all used swelling media, the maximum swelling index was obtained for the media with pH 7.4. On the basis of this observation, it can be concluded that it is recommended to use media with pH 7.4 for the in vitro disintegration of microspheres. 

**Figure 6:**
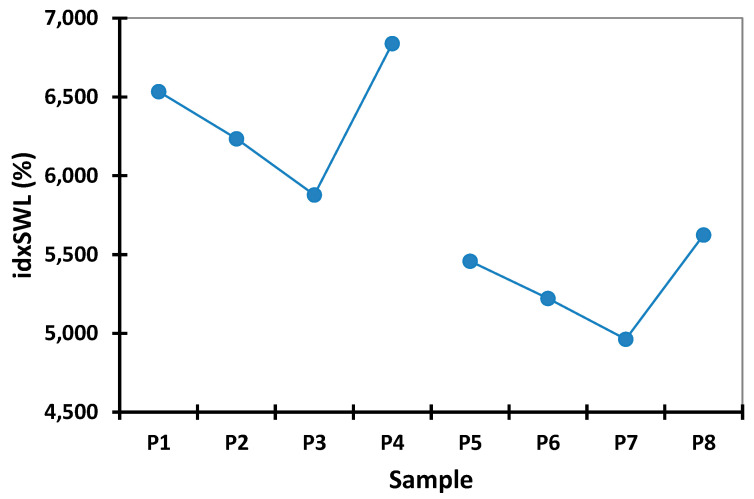
Interval plots of the swelling index (idxSWL (%)) of the microspheres for Sample factor levels.

**Figure 7:**
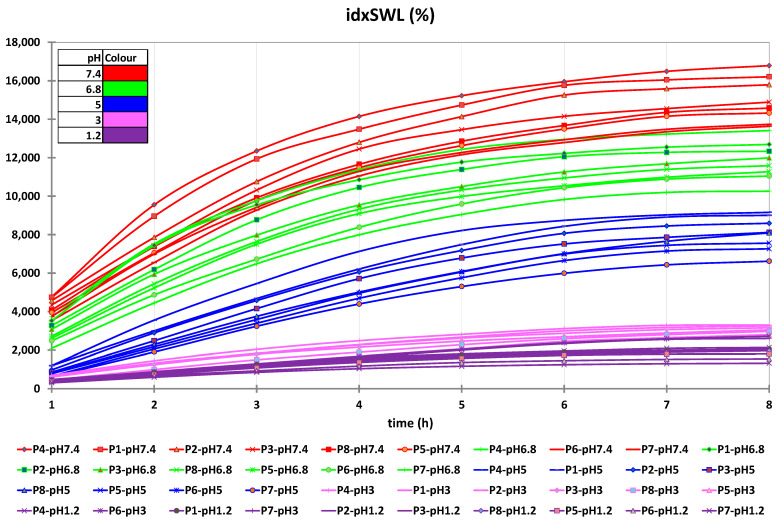
Interval plots of the swelling index (idxSWL (%)) of the microspheres for Sample * pH * Time interaction factor levels.

The microsphere swelling index increases in an inversely proportional manner to the increase of the active drug substance concentration, and in an indirectly proportional manner to the increase of Ch concentration in the microsphere composition. In this context, from the data presented in Tables S4–S12 in the Supplementary Materials, it can be seen that the microspheres without drug content (P4 and P8) present the maximum swelling index in all used media, irrespective of pH. Among them, the samples loaded with drug containing 0.1 g of Ch (P1, P2, and P3) present a higher swelling index than those containing 0.2 g of Ch (P5, P6, and P7), as can be seen from data reported in Figure 6 and Figure 7. 

As a consequence of the increase in the swelling index, the drug release rate in the in vitro test also increases, due to the hydrophilicity of the polymer matrix, which has the ability to absorb water over time, protecting in this way QUE from enzymatic degradation in the gastrointestinal tract [33].

**Figure 8:**
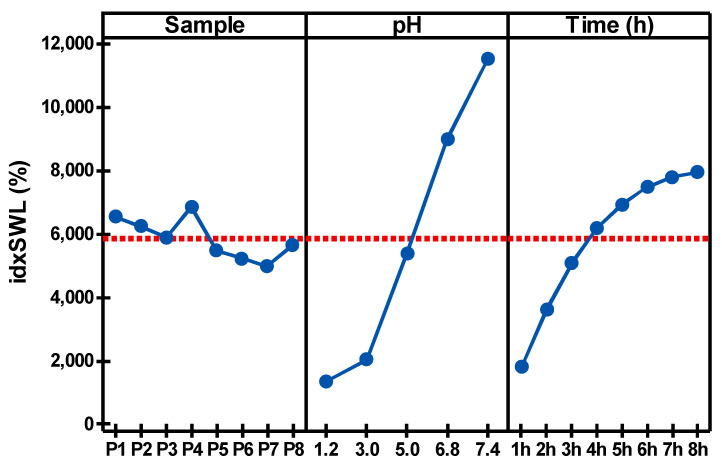
Main effects plot of the three factors (Sample, pH and Time) based on three-way ANOVA (*p* = 0.05) for the microsphere swelling index (idxSWL (%)).

Figure 8 shows a comparison of the factor effects (Sample, pH and Time) on the microspheres’ swelling index (idxSWL (%))based on the three-way ANOVA (*p* = 0.05). It can be seen that the main effect is due to the pH factor, and mainly occurs for the pH values 6.8 and 7.4. This is followed by the Time factor, which has the second most significant effect on the idxSWL parameter; the idxSWL value at 8 h is almost the same as the value at pH = 6.8 (N.B., these values are overall means over the other factor levels). As a consequence consequence, the authors chose to perform the quercetin in vitro release (mQrel (%)) benchmark only for the samples at pH = 7.4.

Some other authors have determined the swelling index of similar chitosan microspheres, but only in a medium at a single pH (7.4), and they obtained swelling indexes of 3568.2 ± 250.2% and 850.5 ± 5.1%, respectively [39,40]. In comparison, in the present study, a swelling index of 16,203 ± 3.2% was obtained.

### 4.3. In Vitro Release Studies of QUE Entrapped in Microspheres

**Figure 9:**
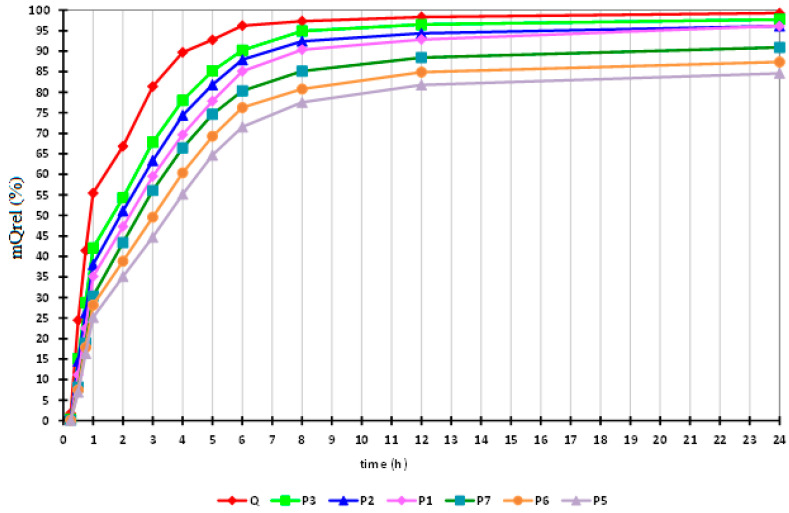
Interval plots of the percentage of QUE released (mQUErel (%)) from the microspheres for Sample * Time interaction factor levels.

In vitro release tests of microspheres loaded with QUE were performed in phosphate buffer solutions with pH 7.4 used as a dissolution medium, which affects the Ch and Na-Alg molecules by deprotonating them, leading to breaks in the Ch-Na-Alg intramolecular bonds concomitant with Alg carboxyl groups ionization. QUE release takes place due to the disintegration of the microsphere, which occurs due to Na-Alg deprotonation at intestinal physiological pH [41]. In other words, at a pH of 7.4, the microspheres have the ability to swell to their maximum size, break, and release the entire amount of entrapped QUE within 24 h. At the same time, if uniform and rapid hydration of microspheres occurs, the gel barrier forms a uniform layer, at which point the diffusion process of QUE takes place. The results of the in vitro release study of QUE from the microspheres are shown in Table S13 in the Supplementary Materials and in Figure 9. 

On the basis of the data depicted in Table 4, it can be observed that QUE release exhibits the highest values when the release tests were carried out using microspheres containing 0.1 g of Ch (samples designated P1, P2, and P3). In the case of samples P5, P6, and P7, which contained 0.2 g of Ch, it can be observed that the QUE release has the lowest values. For the samples in which QUE was used in a concentration of 0.1 g (P3, P7), the active substance was released in percent of 62% and respectively 53%, followed by samples with 0.075 g QUE (P2, P6), which released 60% and 50% of the entrapped QUE, and the samples with 0.050 g QUE (P1, P5), which released 57% and 46% of the entrapped QUE.

**Figure 10:**
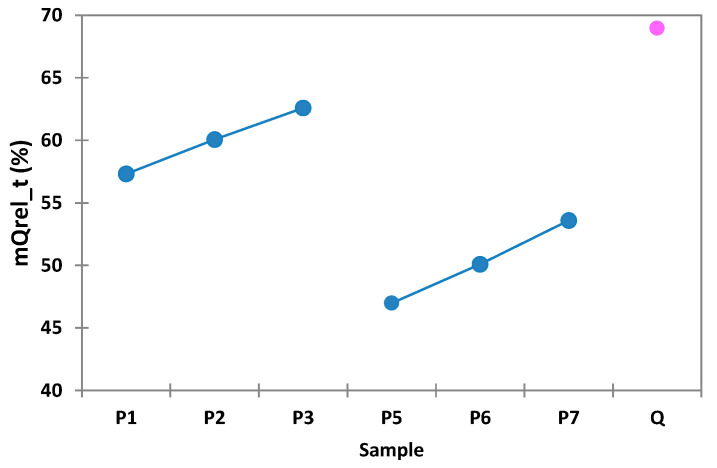
Interval plots of the percentage of QUE released (mQrel (%)) from the microspheres for the Sample factor levels, the pink point corresponds to the Q molecule.

By comparing the experimental data presented in Figure 10, it can be concluded that the highest percentage of QUE release during in vitro tests was obtained in the case where the microspheres had the lowest Ch content and the highest QUE content. From this, it can be concluded that the QUE release during in vitro tests increases in an inversely proportional manner with Ch content and in a directly proportional manner with active drug substance content.

**Figure 11:**
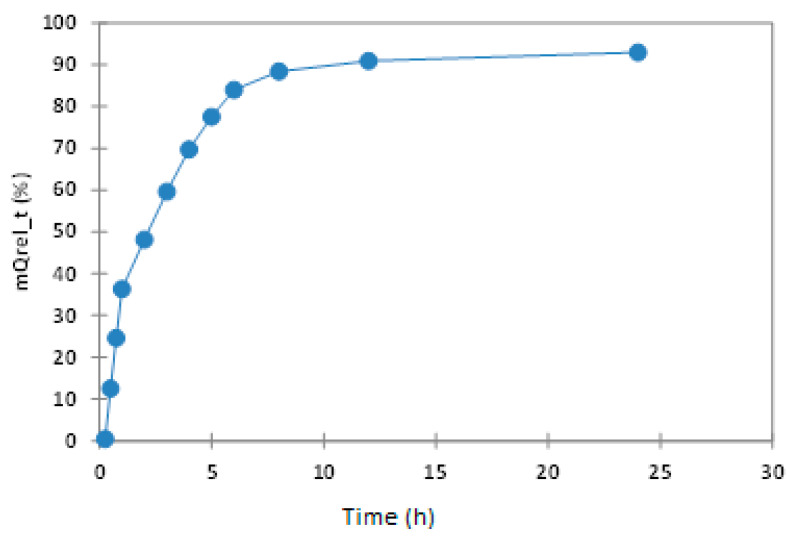
Interval plots of the percentage of QUE released (mQUErel (%)) from the microspheres for Time factor levels.

The experimental data obtained are presented in Table S14 in the Supplementary Materials and in Figure 11, and confirm that in all samples, more than 36% of the active substance was released in first hour, with this quantity reaching 69% after 4 h, and around 88.35% of entrapped QUE having been released after 8 h.

The in, vitro release curve of QUE in phosphate buffer is illustrated in Figure S8 in the Supplementary Materials, and has a different curve from the one observed in the case of microspheres due to the good dissolution of QUE into the phosphate buffer solution. Thus, the in vitro QUE release is directly proportional to the increase of the speed of the QUE dissolution process in the phosphate buffer at pH 7.4. In the case of the newly prepared microspheres, homogenous dispersion of Q determined good control of the dissolution rate into the phosphate buffer. Such systems will release the substance by diffusion as a result of matrix swelling and matrix dissolution/erosion at the periphery of the microcapsules when exposed to the dissolution medium [41]. 

The release of QUE from microspheres in a phosphate buffer solution at pH 7.4 can be controlled by increasing the Ch concentration in the polymeric matrix. Increasing the Ch concentration prevents the erosion of the matrix at the gastric stage, resulting in the controlled release of QUE into the small intestine. These preliminary results are important for the further preparation of new pharmaceutical products, the pharmacological activity of which will be evaluated by in vivo experiments carried out on laboratory animals.

### 4.4. Thermal Analysis of Quercetin Microspheres

**Figure 12:**
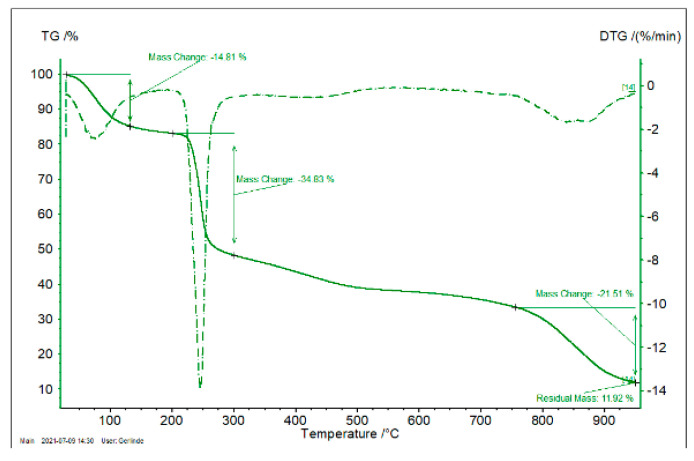
TG and DTG for Na-Alg.

From the thermograms obtained for all QUE microspheres is possible to observe the existence of several stages of degradation of the Q, Na-Alg, Ch and the obtaining microspheres (Figure 12 and Figure S2 from Supplementary Materials).

From Figure 12 (TG obtained for sample Na-Alg), the presence of three different mass losses can be observed, with the first being of 14.81% and associated with water loss (between 20 and 130 °C). Further increase of the temperature leads to a second mass loss of 30.83% associated with the thermal decomposition of Na-Alg (between 200 and 300 °C), followed by one more thermal decomposition with a mass loss of 21.5% (between 750 and 950 °C). Finally, after thermal decomposition, a residue of 11.92% was obtained. In the case of QUE, two mass losses can be observed: the first, at between 20 and 130 °C, is associated with water loss (2.99%), and the second one, taking place between 300 and 400 °C (38.84%), is associated with the thermal decomposition of QUE. This decomposition continues until 950 °C, at which point a residue of 29.49% is finally obtained. In the case of sample P1, six mass losses were observed, with the first of them (between 20 and 120 °C) being associated with water loss (mass loss of 13.41%). Between 120 and 850 °C, another five steps associated with thermal degradation of analyzed sample were observed. At end of the thermal cycle, a mass residue of 31.15% was obtained. In the case of sample P2, a first mass loss was observed between 20 and 120 °C, which is associated with water loss. Furthermore, between 120 and 830 °C another five domains associated with the thermal decomposition of the studied sample were observed. At the end of thermal decomposition at 950 °C a final residue of 29.46% was obtained. In the case of sample P3, a first mass loss of 8.15% was observed between 20 and 120 °C, associated with water loss. Between 120 and 830 °C, another five mass loss domains associated with thermal decomposition of the analyzed sample were observed, and at end of the thermal decomposition cycle, a final residue of 34.09% was observed. For sample P4, a mass loss of 7.80% due to water losses was observed between 20 and 120 °C. Between 120 and 830 °C, the presence of four thermal decomposition domains could be observed, and at end of the thermal cycle a final residue of 26.28% was obtained. Similarly, for sample P5, a water loss of 18.16% was observed (between 20 and 120 °C). Likewise, from 120 to 830 °C, the presence of five decomposition domains was observed. At the end of thermal decomposition, a mass residue of 28.32% was obtained. In the case of sample P6, a water loss of 7.62% was observed, and likewise five thermal decomposition steps between 120 and 830 °C were observed, resulting in a final residue of 33.14%. For sample P7, the water loss was about 5.57%, and likewise, five degradation steps were observed between 120 and 830 °C, with the final residue obtained being 35.31%. In the case of the last sample (P8), a water loss of 3.35% was observed, followed by degradation steps between 120 and 820°, resulting in a final residue of 29.25%. On the basis of the obtained experimental data, it can be concluded that the prepared microspheres are stable and can be used at normal physiological temperature; therefore, such microspheres can be used as drug delivery system.

### 4.5. Scanning Laser Confocal Microscopy Analysis

**Figure 13:**
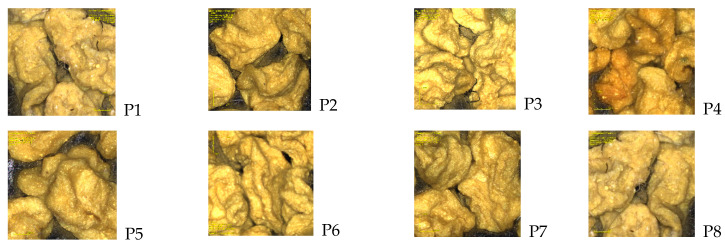
3D laser microscopy of quercetin microspheres.

**Figure 14:**
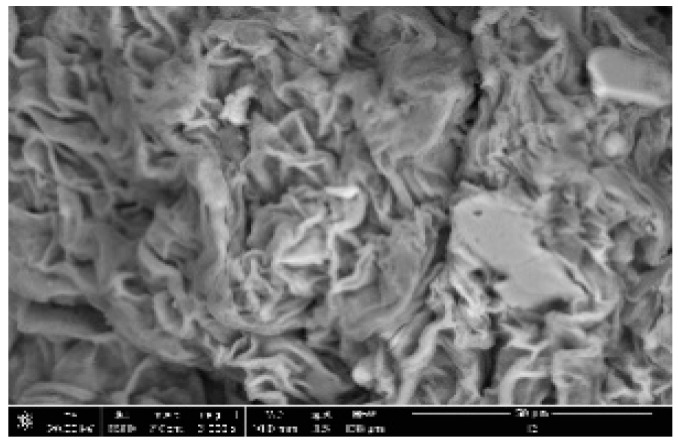
SEM micrograph recorded for sample P1.

The roughness, height, and valley values calculated from the obtained images presented in Figure 13 and Figure 14 [Average roughness (Sa), Mean Square Root Roughness (Sq), Maximum peak height (Sp), Maximum valley depth (Sv), Maximum peak-to valley height (Sz)] are shown in Table 5. All of the aforementioned parameters are presented as average values calculated by a specific formula [42].

All samples exhibited rugosity values (referring to the Sa and Sq) between 20 and 37 µm in the case of Sa, and between 42 and 82 µm in the case of Sq. Both Sa and Sq were used to express the roughness of the material, with the difference between them being the calculation formula. The highest roughness values were registered in the case of samples P8 and P4, which were obtained for the microspheres without any active substance incorporated. Furthermore, the highest rugosity corresponded to samples P4 and P6. The discrepancy between the highest roughness values and Sy are possibly due to the discontinuance of high values on the analyzed area. An example in this matter is the sample P1, whereas the Sy value is much smaller than in the sample P3. This could mean that extremely large heights, in the case of sample P3, are low in number, while the largest height values in sample P1 are more consistent. This also occurs due to the more or less wrinkled analyzed areas. It should also be mentioned that due to the nature of this analysis, while only local aspects are measured, the images of the specific heights measured and the general aspect of the images should also be considered. However, with the larger differences in the present values, the more distinguishing results will be more correct. As for the appearance of the samples, all samples have a similar aspect, with wrinkled areas on the surface of the analyzed granules. Some samples present a smoother surface, which is visible in the analyzed height profile for each sample.

### 4.6. Results of Scanning Electron Microscopy (SEM)

The SEM images recorded for sample P1 are presented in Figure 14, and the SEM images recorded for the remaining samples are presented in Figure S3 in the Supplementary Materials. Analyzing the pictures presented in the figure, it can be observed that sodium alginate exhibits a compact structure, QUE has a fibrous structure, and Ch presents a multi-layer structure.

The obtained microspheres exhibit a relatively compact structure, with a high number of folds, and some crevasses located between these folds. Additionally, the presence of some pores can be observed on the superficial layer, meaning that such microspheres are able to achieve controlled release for the entrapped (quercetin) active drug.

### 4.7. Statistical Analysis

#### 4.7.1. Nonlinear Regression of the Swelling Index and In Vitro Release Time Series

Considering the fact that the swelling index and the quercetin in vitro release are measured again time, they can be considered as time series and can be fitted against the Time factor levels. A single exponential function—Y = Ymax × (1 − exp(−K × X))—was used as the nonlinear fitting function. The parameter Ymax represents the asymptotic maximum that the idxSWL and mQrel can achieve; K is a constant, with a positive value constraint; and the value H = 0.69/K was calculated, denoting the “half-life”.

**Figure 15:**
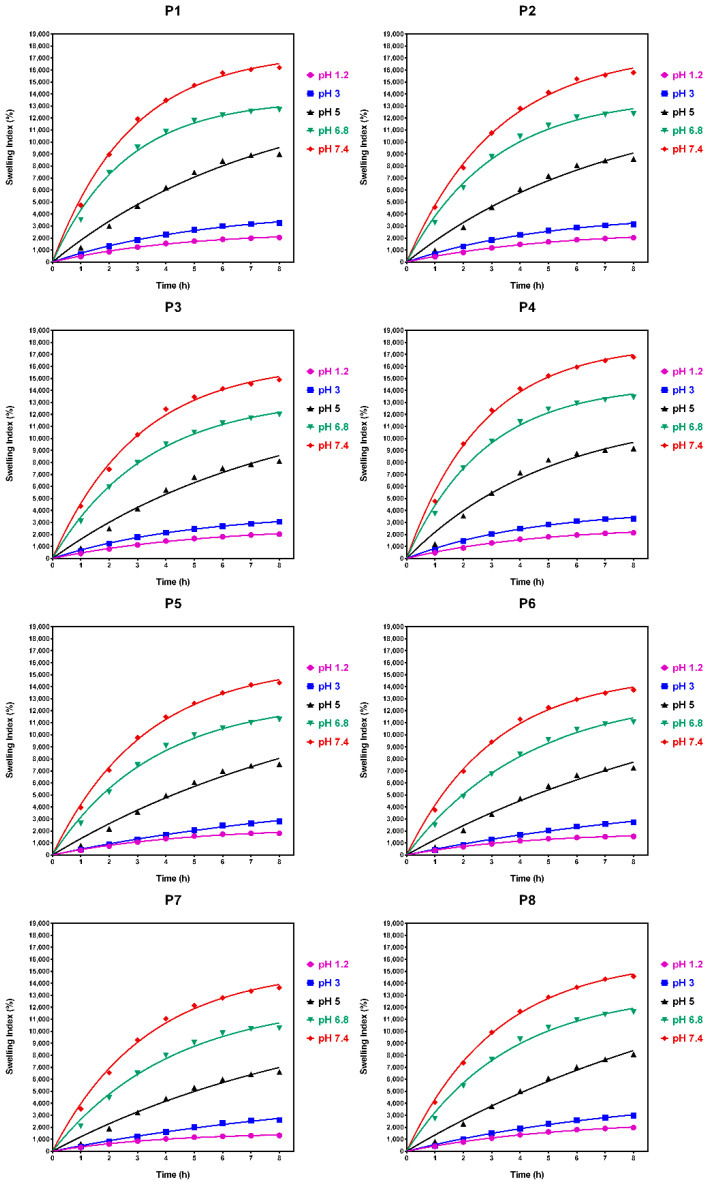
Graphical comparisons of the results of the nonlinear regression of the idxSWL (%) time series for samples P1–P8 and all pH factor levels.

The data obtained for the nonlinear fitting results of the idxSWL (%) time series for samples P1–P8 and all pH factor levels are presented in Tables S15–S22 in the Supplementary Materials. Figure 15 presents graphical comparisons of the results of the nonlinear regression of the idxSWL (%) time series for samples P1–P8 and all pH factor levels.

The determination coefficient, R^2^ (i.e., R square), for all of the samples (P1–P8) and for all pH levels achieved values higher than 0.96 (data depicted in Table S23 in the Supplementary Materials). This fact demonstrates the high accuracy of the analytical measurements, and that the chosen exponential fitting accurately describes the time evolution of the swelling index for all samples and for all pH levels. Additionally, this fact demonstrates the high accuracy of the analytical measurements, and that the chosen exponential fitting accurately describes the time evolution of the quercetin in vitro release for all studied samples.

#### 4.7.2. Multivariate Analysis

In Section 2, it was stated that a multivariate statistical sequence was used. This consisted of the PCA, LDA, MANOVA (*p* = 0.05) and AHC statistical methods. The multivariate analysis was performed in order to determine which sample offered the best combined performance in quercetin release and microsphere surfactant roughness. 

To obtain suitable results, it was necessary to generate the proper number, and thus the composition, of microsphere clusters. After the clustering of the microsphere samples, the PCA results can be correctly interpreted.

Another important aspect of the multivariate statistical analysis is that only the MANOVA has a statistical significance level (*p* = 0.05) that gives statistical confidence its results, and thus the validity of any conclusions drawn. 

The multivariate statistical sequence begins with the PCA method, which generates the principal coordinates (PC1–PC11) of the studied parameters for all of the microspheres (i.e., the QUE and Ch combinations). The principal coordinates PC1 to PC2 cover 98.99% of the cumulative explained variance. 

**Figure 16:**
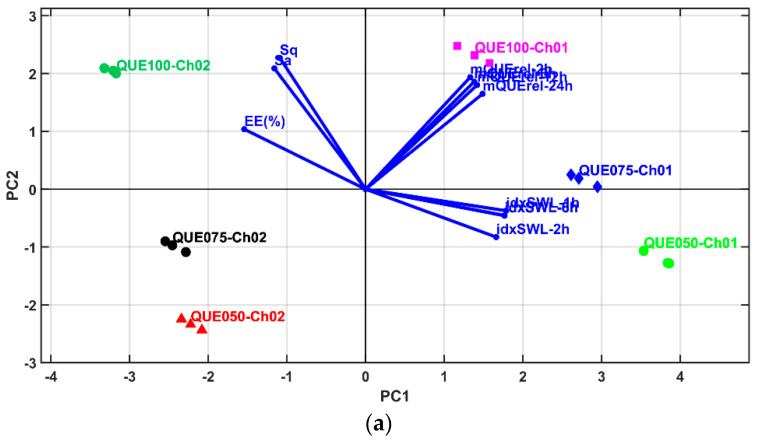

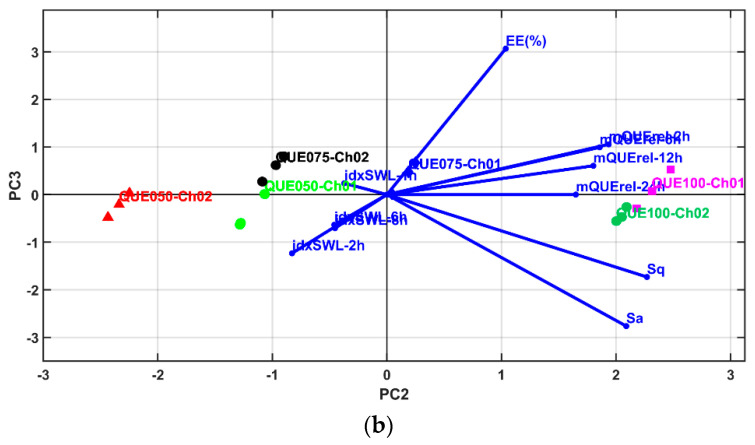
Biplots of PCA results: (**a**) 2D representation for PC1 and PC2 principal components; (**b**) 2D representation for PC2 and PC3 principal components.

**Figure 17:**
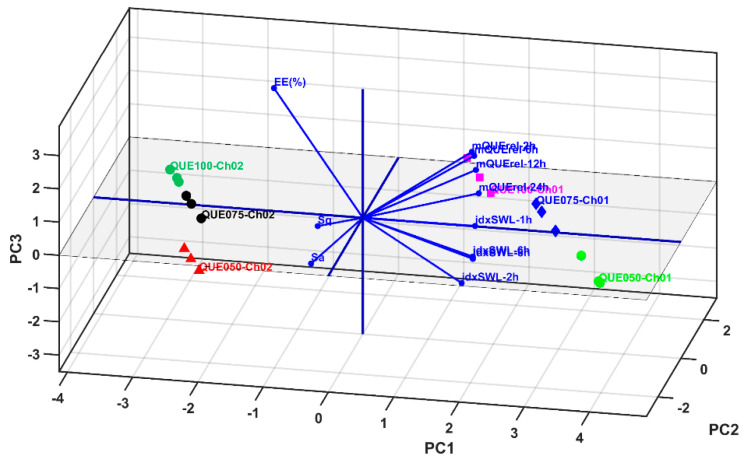
Biplot of PCA results in 3D representation for PC1, PC2 and PC3 principal components.

The PCA 2D and 3D biplots (Figure 16a,b and Figure 17) display the variable vectors, indicating the direction in which the corresponding variables have the highest abundance or concentration values. Additionally, the samples are also displayed; thus, in this way, PCA consists of an ordination multivariate statistical method that generates relative comparisons between the studied samples on the basis of their parameter values. Furthermore, the initial idxSWL and mQUErel Sample * Time levels was shortened to mQUErel_2 h, mQUErel_6 h, mQUErel_12 h, mQUErel_24 h, idxSWL_1 h, idxSWL_2 h, idxSWL_6 h, idxSWL_8 h. The reason for this was the presence of strong correlations between Time levels, and for each parameter; thus, redundant information could corrupt the PCA biplots, scores, and loadings. 

The principal coordinates corresponding to PC1–PC11 were used as the numerical inputs for the MANOVA (*p* = 0.05) test method. The results obtained using this method were: a matrix of the statistical significances of the pairwise sample comparisons (Bonferroni corrected values, *p* = 0.05) and the canonical coordinates (Canon1–Canon11) of the variables for all the microsphere samples. 

**Figure 18:**
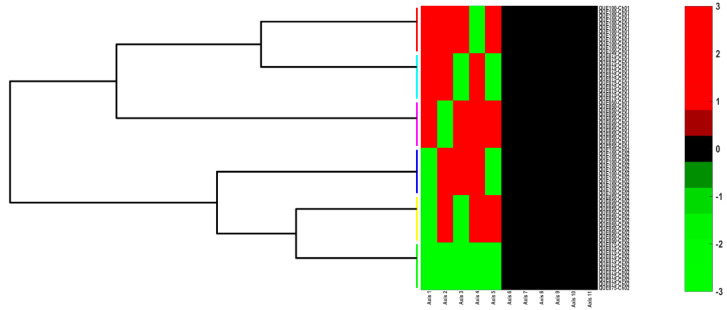
Cluster analysis of MANOVA (*p* = 0.05) results: Heatmap representation for PC1 ÷ PC11 principal coordinates. Different colors for groupings of horizontal lines denotes a sample cluster.

**Figure 19:**
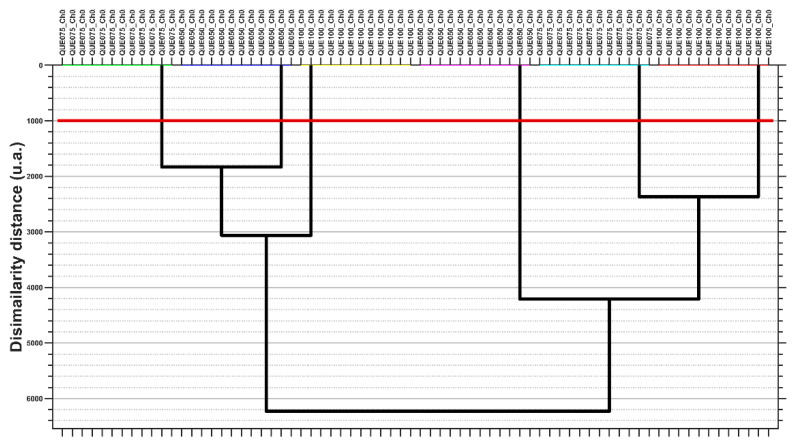
Cluster analysis of MANOVA (*p* = 0.05) results: dendrogram representation for principal coordinates of PC1 ÷ PC11 with the cut-off red line used to generate the clusters. Different colors for groupsing of horizontal lines denote sample clusters.

To obtain the microsphere clusters, the AHC clustering method was used on the coordinate values of Canon1 to Canon11. The AHC results are presented as heat maps with a dendrogram (Figure 18), and a simple dendrogram with the cut-off similarity distance value (Figure 19), which were used to obtain an appropriate number of clusters on the basis of the MANOVA *p*-values matrix.

The PCA heat map representation emphasizes that the principal coordinates of PC1–PC5 present significantly high values that are able to be used to generate an appropriate number of clusters using the AHC method.

**Figure 20:**
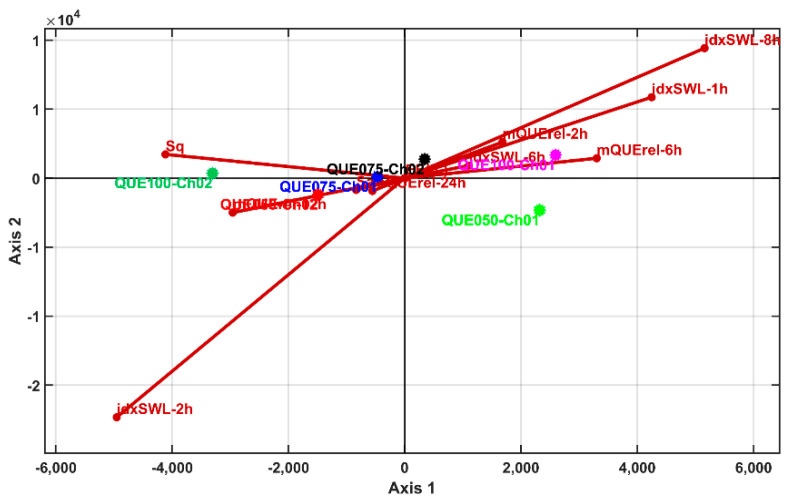
Biplots of LDA results in 2D representation for Axis1 and Axis2 canonical components.

**Figure 21:**
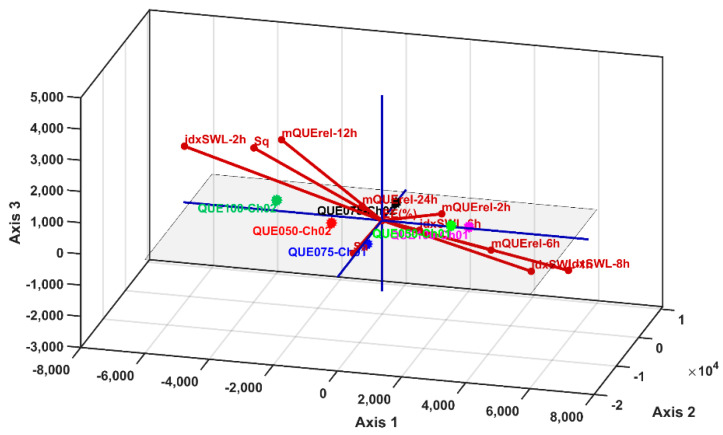
Biplot of LDA results in 3D representation for Axis1, Axis2 and Axis3 canonical components.

Linear discriminant analysis was performed for the selected variables (like the PCA) and the AHC method was applied to generate a heatmap and clustering dendrogram. The results were similar to those obtained with PCA. Figure 20 and Figure 21 show the 2D and 3D LDA biplots and show the similarity with the PCA biplots. As a consequence, the LDA heatmap and LDA clustering dendrogram are not shown, but rather the AHC with the results of LDA canonical coordinate clustering (which is the same as with the PCA coordinates). The PCA/LDA clustering results prescribe that all samples levels are singleton clusters—meaning that they are statistically significantly different based on their multivariate profile.

At this point, an accurate comparative analysis can be performed between the sample levels (i.e., microsphere samples) using the PCA biplots (Figure 16 and Figure 17).

Samples QUE100_Ch01 (P3) and QUE100_Ch02 (P7) present the highest values of roughness (highest values for Sa and Sq (µm)), EE(%) and mQUErel (%). Furthermore, sample QUE100_CH01 (P3) has higher swelling index values than QUE100_Ch02, but lower than those of QQUE050_Ch01 and QUE075_Ch01. Despite this fact, the latter samples have higher values of mQrel than QUE100_Ch02, QUE075_Ch02 and QUE050_Ch02, but lower values for EE(%), Sa and Sq parameters. 

In conclusion, the best microsphere sample for QUE entrapment, with high values of all parameters is QUE100_Ch01 (P3), which entraps large enough quantities of QUE while offering the highest values of roughness and in vitro release. This fact qualifies this sample for further medical testing.

## 5. Conclusions

Analyzing the data obtained for the QUE entrapment efficiency, it can be observed that the complex coacervation method used for microsphere preparation offers the highest efficiency. Using this method, we obtained microspheres with 86.07% encapsulation efficiency, which was greatly influenced by increased CH concentration. Furthermore, on the basis of the in vitro studies of QUE release in phosphate buffer (pH 7.4) and the microsphere swelling studies in different media (with pH values of 1.2, 3.0, 5.0, 6.8, and 7.4), it can be concluded that the inclusion of polymers in the microsphere matrix influences the QUE release rate. The inclusion of concomitant polymers in the microsphere matrix prevents its erosion and degradation at the gastric stage, resulting in release of QUE into the small intestine. QUE release from the matrix system at the intestinal stage is achieved by diffusion through the pores on the surface of the microspheres, which were observed by SEM analysis. As a consequence of the swelling and erosion processes, the dimensions of the superficial pores will increase, with beneficial consequences for QUE release. Laser confocal microscopy confirmed that the prepared microspheres were rough, similar in appearance, and were wrinkled on the surface, with a relatively compact structure with many folds. From the TG studies, it can be concluded that the prepared microspheres are stable at normal usage temperatures.

Such complex microspheres can be used at normal physiological temperatures as carrier/drug delivery systems. On the basis of the statistical analysis, it was observed that the best microsphere sample for QUE entrapment, with high values of all parameters, was QUE100_Ch01 (P3), which entrapped sufficiently large quantities of QUE, with the highest values for roughness and in vitro release. This fact qualifies this sample as being suitable for future evaluation of living cell activity. Therefore, we consider further investigations necessary in order to evaluate the biological effects of microspheres with QUE, and the manner in which endothelial cells activate to maximize their benefits for the protection of living organisms.

In other words, the present study suggests that QUE, which is a water-insoluble active substance the presents large problems with respect to its stability in the face of external factors, can be successfully used as a drug by microencapsulation in modern pharmaceutical formulations, such as microspheres.

## Figures and Tables

**Table 1:** Formulas of prepared quercetin microspheres.

Samples	The Mass of Quercetin (g)	The Mass of Chitosan (g)	The Mass pf Sodium Alginate (g)
P1 (QUE050_Ch01)	0.050	0.1	0.75
P2 (QUE075_Ch01)	0.075	0.1	0.75
P3 (QUE100_Ch01)	0.100	0.1	0.75
P4	-	0.1	0.75
P5 (QUE050_Ch02)	0.050	0.2	0.75
P6 (QUE075_Ch02)	0.075	0.2	0.75
P7 (QUE100_Ch02)	0.100	0.2	0.75
P8	-	0.2	0.75

**Table 2:** QUE EE (%) from microspheres.

Sample	EE (%)
P1	82.217d ± 0.668
P2	83.257c, d ± 0.609
P3	84.047b, c ± 0.764
P5	84.180b, c ± 0.478
P6	85.227a, b ± 0.502
P7	86.067a ± 0.276

Note: Different letters (a, b, c, d) following the means indicate statistically significant means. Results were calculated with post hoc Duncan (*p* = 0.05) multiple comparisons test, within the two-way ANOVA test (*p* = 0.05). An interval plot of the microsphere entrapment efficiency is presented in Figure S1 in the Supplementary Materials.

**Table 3:** Percentage of swelling index (idxSWL (%)) for microspheres as a function of pH factor level.

pH	idxSWL (%)
1.2	1327.290e ± 529.110
3	2020.404d ± 864.895
5	5375.740c ± 2583.403
6.8	8984.463b ± 3128.500
7.4	11,514.305a ± 3730.353

Note: Different letters following the means indicate statistically significant means. Results were calculated with post hoc Duncan (*p* = 0.05) multiple comparisons test, with the two-way ANOVA test (*p* = 0.05).

**Table 4:** Percentage of QUE released (mQUErel (%)) from the microspheres as a function of the Sample factor levels.

Sample	mQUErel (%)
P1	57.303d ± 32.163
P2	60.056c ± 32.071
P3	62.584b ± 32.569
P5	46.979g ± 28.720
P6	50.084f ± 29.809
P7	53.584e ± 31.223
QUE	68.958a ± 30.654

Note: different letters following the means indicate statistically significant means. Results were calculated with post hoc Duncan (*p* = 0.05) multiple comparisons test, within the two-way ANOVA test (*p* = 0.05).

**Table 5:** Calculated roughness of microspheres.

Sample	Evaluation Area (µm^2^)	Sa (µm)	Sq (µm)	Sp (µm)	Sv (µm)	Sy (µm)
P1	1279 × 1280	20.890h ± 0.0082	42.110h ± 0.0163	472.10h ± 0.02	386.56h ± 0.01	858.67h ± 0.03
P2	1279 × 1280	22.013g ± 0.0170	48.733g ± 0.0205	563.08g ± 0.03	489.47g ± 0.01	1052.56g ± 0.03
P3	1279 × 1280	28.330d ± 0.0408	62.630d ± 0.0163	713.99d ± 0.02	672.91d ± 0.02	1386.90d ± 0.01
P4	1279 × 1280	29.790c ± 0.0082	64.530c ± 0.0163	773.75c ± 0.02	712.11c ± 0.01	1586.90c ± 0.01
P5	1279 × 1280	24.260f ± 0.0163	50.490f ± 0.0408	578.75f ± 0.01	462.30f ± 0.02	1041.05f ± 0.02
P6	1279 × 1280	24.790e ± 0.0082	53.260e ± 0.0163	624.40e ± 0.01	538.86e ± 0.01	1165.51e ± 0.02
P7	1279 × 1280	34.560b ± 0.0408	74.020b ± 0.0082	710.85b ± 0.02	540.86b ± 0.02	1249.72b ± 0.01
P8	1279 × 1280	36.760a ± 0.0408	82.180a ± 0.0082	776.42a ± 0.02	577.15a ± 0.01	1359.72a ± 0.01

## Data Availability

The data presented in this study are available on request from the corresponding author.

## Supplementary Materials

The following supporting information can be downloaded at: https://www.mdpi.com/article/10.3390/polym14030490/s1, Figure S1. Interval plots of the microspheres’ entrapment efficiency (EE(%)) for sample factor levels; Figure S2. TG and DTG for: a—Na-Alg, b—QUE, c—Ch, d—P1, e—P2, f—P3, g—P4, h—P5, i—P6, j—P7, k—P8; Figure S3. SEM analysis of quercetin microspheres; Figure S4. Interactions established during microsphere formation and drug loading; Figure S5. Ch FT-IR spectra; Figure S6. Na-Alg FT-IR spectra; Figure S7. QUE FT-IR spectra; Figure S8. Interval plots of the microspheres’ percentage of Q released (mQrel (%)) from microspheres for Sample * Time interaction factor levels. Table S1. Percentage of swelling index (idxSWL (%)) from microspheres, the results belong to the Time factor levels; Table S2. Percentage of swelling index (idxSWL (%)) from microspheres, the results belong to the Sample*pH interaction factor levels; Table S3. Percentage of swelling index (idxSWL (%)) from microspheres, the results belong to the Sample * Time interaction factor levels; Table S4. Percentage of swelling index (idxSWL (%)) from microspheres, the results belong to the Sample factor levels; Table S5. Percentage of swelling index (idxSWL (%)) from microspheres, the results belong to the P1*pH*Time interaction factor levels; Table S6. Percentage of swelling index (idxSWL (%)) from microspheres, the results belong to the P2*pH*Time interaction factor levels; Table S7. Percentage of swelling index (idxSWL (%)) from microspheres, the results belong to the P3*pH*Time interaction factor levels; Table S8. Percentage of swelling index (idxSWL (%)) from microspheres, the results belong to the P4*pH*Time interaction factor levels; Table S9. Percentage of swelling index (idxSWL (%)) from microspheres, the results belong to the P5*pH*Time interaction factor levels; Table S10. Percentage of swelling index (idxSWL (%)) from microspheres, the results belong to the P6*pH*Time interaction factor levels; Table S11. Percentage of swelling index (idxSWL (%)) from microspheres, the results belong to the P7*pH*Time interaction factor levels; Table S12. Percentage of swelling index (idxSWL (%)) from microspheres, the results belong to the P8*pH*Time interaction factor levels; Table S13. Percentage of QUE released (mQrel (%)) from microspheres, the results belong to the Sample * Time interaction factor levels; Table S14. Percentage of QUE released (mQUErel (%)) from microsphere, the results belong to the Time interaction factor levels; Table S15. Results of the non-linear regression of the idxSWL (%) time series for sample P1 and all pH factor levels; Table S16. Results of the non-linear regression of the idxSWL (%) time series for sample P2 and all pH factor levels; Table S17. Results of the non-linear regression of the idxSWL (%) time series for sample P3 and all pH factor levels; Table S18. Results of the non-linear regression of the idxSWL (%) time series for sample P4 and all pH factor levels; Table S19. Results of the non-linear regression of the idxSWL (%) time series for sample P5 and all pH factor levels; Table S20. Results of the non-linear regression of the idxSWL (%) time series for sample P6 and all pH factor levels; Table S21. Results of the non-linear regression of the idxSWL (%) time series for sample P7 and all pH factor levels; Table S22. Results of the non-linear regression of the idxSWL (%) time series for sample P8 and all pH factor levels; Table S23. Results of the non-linear regression of the QUE in vitro release time series for samples P1-P7 and QUE.
