# Supplementary material for: Preparation and Characterization of Chitosan-Alginate Microspheres Loaded with Quercetin
Source: Polymers (Basel). 2022 Jan 26;14(3):490. doi: 10.3390/polym14030490 (PMC8839549; doi:10.3390/polym14030490)
Supplement: Supplementary file 1 [file polymers-14-00490-s001.zip › polymers-1530097-supplementary.pdf]

polymers

Article

Preparation and Characterization of Chitosan-Alginate Microspheres Loaded with Quercetin

Frenț Olimpia Daniela [1], Duteanu Narcis [2,*], Teusdea Alin Cristian [3,*], Ciocan Stefania [2,*], Vicaș Laura [4,*], Jurca Tunde [4], Muresan Mariana [5,†], Pallag Annamaria [4], Ianasi Paula [6,†] and Marian Eleonora [4,†]

[1] Doctoral School of Biomedical Science, University of Oradea, 1 University Street, 410087 Oradea, Romania; daniela.olimpia@yahoo.com
[2] Faculty of Industrial Chemistry and Environmental Engineering, Politehnica University of Timisoara, 2 Piata Victoriei, 300006 Timisoara, Romania
[3] Faculty of Environmental Protection, University of Oradea, 26th Gen. Magheru Avenue, 410087 Oradea, Romania
[4] Department of Pharmacy, Faculty of Medicine and Pharmacy, University of Oradea, 29 Nicolae Jiga Street, 410028 Oradea, Romania; jurcatunde@yahoo.com (J.T.); annamariapallag@gmail.com (P.A.); marian_eleonora@yahoo.com (M.E.)
[5] Department of Preclinical Discipline, Faculty of Medicine and Pharmacy, University of Oradea, 10, 1th December Square, 410068 Oradea, Romania; marianamur2002@yahoo.com
[6] INCEMC-National Institute for Research and Development in Electrochemistry and Condensed Matter—Timisoara, No.144 Dr. A. Paunescu Podeanu Street, 300569 Timisoara, Romania; paulasvera@gmail.com
* Correspondence: narcis.duteanu@upt.ro (D.N.); ateusdea@yahoo.co.uk (T.A.C.); ciocanstefania10@gmail.com (C.S.); laura.vicas@gmail.com (V.L.)
† These authors contributed equally to this work.

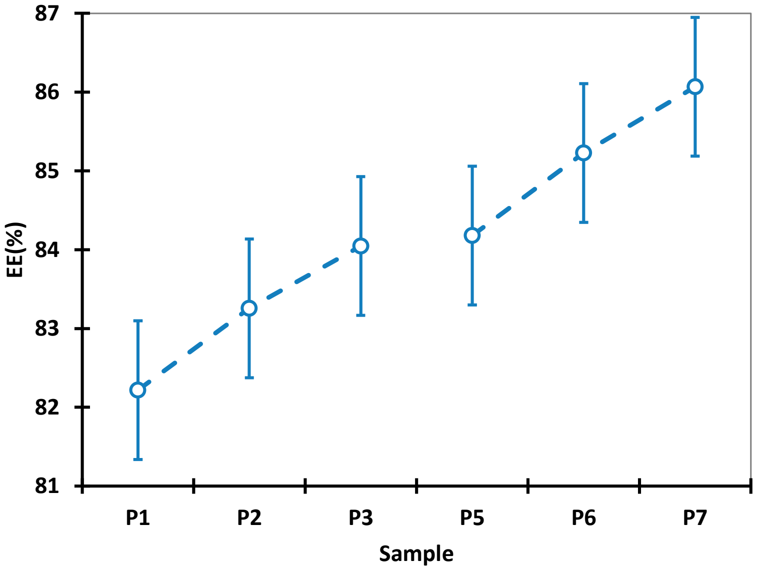

Supplementary Figure S1. Interval plots of the microspheres' entrapment efficiency (EE(%)) for sample factor levels

Supplementary Table S1. Percentage of swelling index (idxSWL (%)) from microspheres

The results belong to the Time factor levels

Time	idxSWL (%)
**1h**	1797.265h ±1550.706
**2h**	3603.814g ±2826.160
**3h**	5063.423f ±3736.602
**4h**	6165.873e ±4319.882
**5h**	6922.697d ±4663.962
**6h**	7470.614c ±4892.880
**7h**	7790.508b ±5021.012
**8h**	7941.329a ±5082.533

Note: Different letters that follow the means prescribe statistically significant means. Results were calculated with post-hoc Duncan ($P = 0.05$) multiple comparisons test, within the two-way ANOVA test ($P = 0.05$).

Supplementary Table S2. Percentage of swelling index (idxSWL (%)) from microspheres
The results belong to the Sample*pH interaction factor levels

pH	P1	P2	P3	P4	P5	P6	P7	P8
**1.2**	1473.425ah ±538.853	1427.960ai ±539.484	1404.273aj ±542.794	1528.835ag ±564.444	1307.667al ±496.099	1130.668am ±402.629	979.569an ±343.418	1365.924ak ±528.571
**3**	2275.329z ±881.996	2218.219aa ±843.629	2112.933ab ±794.380	2401.298y ±882.953	1783.587ad ±800.941	1745.538ae ±788.417	1690.836af ±778.962	1935.494ac ±841.999
**5**	6113.748r ±2723.801	5847.415s ±2622.650	5435.710t ±2514.804	6561.800q ±2730.620	4945.600v ±2379.324	4701.554w ±2306.529	4312.221x ±2065.922	5087.868u ±2456.338
**6.8**	10074.148j ±2983.065	9590.240k ±3099.716	8993.680l ±2941.007	10548.054g ±3193.720	8401.038n ±2888.310	8052.005o ±2917.265	7544.502p ±2795.821	8672.039m ±2982.315
**7.4**	12730.665b ±3802.067	12091.470c ±3822.017	11447.610d ±3564.082	13154.598a ±3894.450	10855.442f ±3475.770	10481.360h ±3318.026	10291.154i ±3382.006	11062.138e ±3486.226

Note: Different letters that follow the means prescribe statistically significant means. Results were calculated with *post-hoc* Duncan (P = 0.05) multiple comparisons test, within the two-way ANOVA test (P = 0.05).

Supplementary Table S3. Percentage of swelling index (idxSWL (%)) from microspheres
The results belong to the Sample*Time interaction factor levels

Time (h)	P1	P2	P3	P4	P5	P6	P7	P8
1	2111.586bb ±1708.746	1977.970bc ±1648.481	1863.390bd ±1570.016	2173.230ba ±1740.397	1636.347bf ±1415.783	1533.027bg ±1355.169	1390.005bh ±1252.721	1692.562be ±1465.031
2	4314.516as ±3284.754	3809.003au ±2761.908	3576.851av ±2638.858	4588.448aq ±3402.536	3218.112ax ±2506.902	3089.102ay ±2459.149	2858.531az ±2297.026	3375.949aw ±2606.158
3	5847.523ai ±4227.424	5419.997al ±3776.339	5071.436an ±3551.664	6180.719ag ±4299.870	4646.097ap ±3450.504	4349.597ar ±3266.790	4211.661at ±3227.409	4780.357ao ±3462.475
4	6876.459y ±4674.870	6611.087ab ±4437.5961	6259.761ae ±4227.238	7348.459r ±4879.085	5716.531aj ±4009.670	5443.489ak ±3892.903	5218.928am ±3817.495	5852.271ai ±4053.975
5	7680.065m ±5036.429	7400.024q ±4833.197	6975.384w ±4538.154	8097.092h ±5232.432	6458.980ac ±4331.477	6204.656af ±4222.779	5935.447ah ±4170.873	6629.931aa ±4399.728
6	8261.844g ±5295.406	8019.329i ±5159.513	7485.099p ±4768.962	8541.580d ±5423.352	7040.053v ±4540.720	6771.981z ±4448.116	6440.157ad ±4374.256	7204.868t ±4613.712
7	8534.763e ±5368.766	8265.205g ±5224.243	7787.080 ±4876.678	8824.191b ±5550.553	7402.509q ±4742.855	7123.249u ±4614.031	6766.793z ±4533.979	7620.270n ±4807.913
8	8640.945c ±5406.801	8377.873f ±5256.6826	8011.727j ±4971.906	8957.618a ±5649.230	7550.705o ±4800.800	7262.703s ±4678.650	6887.728x ±4609.238	7841.332k ±4850.132

Note: Different letters that follow the means prescribe statistically significant means. Results were calculated with *post-hoc* Duncan (P = 0.05) multiple comparisons test, within the two-way ANOVA test (P = 0.05).

Supplementary Table S4. Percentage of swelling index (idxSWL (%)) from microspheres

The results belong to the Sample factor levels

Sample	idxSWL (%)
**P1**	6533.463b ±5033.793
**P2**	6235.061c ±4838.292
**P3**	5878.841d ±4554.464
**P4**	6838.917a ±5206.396
**P5**	5458.667f ±4376.188
**P6**	5222.225g ±4251.799
**P7**	4963.656h ±4154.492
**P8**	5624.693e ±4448.765

Note: Different letters that follow the means prescribe statistically significant means. Results were calculated with *post-hoc* Duncan (P = 0.05) multiple comparisons test, within the two-way ANOVA test (P = 0.05)

Supplementary Table S5. Percentage of swelling index (idxSWL (%)) from microspheres

The results belong to the P1*pH*Time interaction factor levels

Time (h)	P1_pH1.2	P1_pH3	P1_pH5	P1_pH6.8	P1_pH7.4
1	456.777ic ±0.424	645.287hw hx ±0.525	1198.023he ±0.469	3521.980eq ±1.367	4735.863ec ±0.719
2	856.900hp ±0.699	1323.027ha ±0.555	2997.160fb ±0.497	7442.653cv ±95.926	8952.840ca ±0.537
3	1246.487hd ±0.755	1832.407gj ±0.390	4679.867ee ±4.621	9547.803br ±0.600	11931.053ap ±2.901
4	1547.333gt ±0.396	2292.380fu fv ±0.933	6209.833dm ±1.601	10853.383bd ±0.978	13479.363uv ±0.882
5	1741.553go ±0.681	2676.103fj ±0.792	7480.103cu ±1.104	11767.100aq ±0.940	14735.467l ±0.685
6	1894.560gh ±0.479	2998.780fb ±0.455	8430.207cg ±1.576	12225.417al ±1.046	15760.257g ±1.038
7	1995.650ge ±0.540	3175.783ew ±0.568	8910.327cb ±1.129	12547.567af ±1.489	16044.487d ±1.790
8	2048.137gb ±0.830	3258.867eu ± 27.955	9004.460bz ±1.263	12687.277ad ±1.079	16205.987c ±1.913

Note: Different group letters that follow the means prescribe statistically significant means. Results were calculated with *post-hoc* Duncan (P = 0.05) multiple comparisons test, within the two-way ANOVA test (P = 0.05).

Supplementary Table S6. Percentage of swelling index (idxSWL (%)) from microspheres
The results belong to the P2*pH*Time interaction factor levels

Time (h)	P2_pH1.2	P2_pH3	P2_pH5	P2_pH6.8	P2_pH7.4
**1**	435.303id ±1.211	631.933hx hy ±0.875	978.100hl ±0.829	3275.893et ±1.271	4568.620ef ±1.081
**2**	806.050hq ±1.315	1302.777hb ±0.964	2896.567fd ±1.162	6185.953dn ±1.099	7853.670co ±2.227
**3**	1175.643hf ±1.505	1826.553gj gk ±0.741	4575.710ef ±0.962	8767.513cc ±1.081	10754.563be ±1.496
**4**	1480.757gw ±0.919	2272.223fw ±1.038	6054.140dp ±0.868	10452.983bh ±1.727	12795.333ac ±1.001
**5**	1684.350gp ±0.671	2621.343fl ±0.942	7174.140cz ±1.148	11385.107av aw ±1.911	14135.180q ±1.288
**6**	1854.713gi ±1.044	2871.620fe ±1.327	8066.483cl ±1.060	12047.663an ±1.161	15256.163i ±1.656
**7**	1958.990gg ±0.703	3063.430fa ±0.516	8447.027cf ±0.940	12275.613ak ±1.806	15580.963h ±2.100
**8**	2027.873gd ±0.753	3155.870ex ±1.506	8587.157ce ±1.076	12331.197aj ±2.122	15787.267f ±2.295

Note: Different group letters that follow the means prescribe statistically significant means. Results were calculated with *post-hoc* Duncan (P = 0.05) multiple comparisons test, within the two-way ANOVA test (P = 0.05).

Supplementary Table S7. Percentage of swelling index (idxSWL (%)) from microspheres

The results belong to the P3*pH*Time interaction factor levels

Time (h)	P3_pH1.2	P3_pH3	P3_pH5	P3_pH6.8	P3_pH7.4
1	416.917ie ±0.601	613.850hz ±0.205	857.050hp ±0.764	3080.260ez ±0.821	4348.873ei ±1.137
2	784.763hr ±1.123	1248.227hd ±0.831	2485.083fp ±0.784	5938.040dr ±0.772	7428.143cw ±0.797
3	1128.477hg ±0.838	1786.767gn ±0.487	4155.423ej ±1.086	7973.410cn ±1.045	10313.103bj ±1.591
4	1448.620gx ±47.773	2153.520fy ±0.694	5711.593dt ±1.114	9536.800br ±1.300	12448.270ag ±0.928
5	1667.210gq ±1.271	2467.757fq ±0.619	6784.837df ±1.317	10498.870bg ±1.250	13458.247w ±0.776
6	1815.150gk gl ±1.066	2693.327fi ±0.876	7514.070cs ±1.150	11255.170ay ±1.539	14147.780q ±1.590
7	1954.657gg ±0.646	2888.133fd ±0.792	7862.407co ±0.996	11681.263ar ±2.068	14548.940n ±2.765
8	2018.393gd ±0.681	3051.880fa ±1.283	8115.217cj ±1.055	11985.623ao ±2.127	14887.520k ±1.954

Note: Different group letters that follow the means prescribe statistically significant means. Results were calculated with *post-hoc* Duncan (P = 0.05) multiple comparisons test, within the two-way ANOVA test (P = 0.05).

Supplementary Table S8. Percentage of swelling index (idxSWL (%)) from microspheres
The results belong to the P4*pH*Time interaction factor levels

Time (h)	P4_pH1.2	P4_pH3	P4_pH5	P4_pH6.8	P4_pH7.4
**1**	474.700ib ±0.403	696.883hu ±0.590	1201.290he ±0.610	3728.180en ±0.885	4765.097eb ±0.872
**2**	874.710ho ±0.764	1448.537gx ±0.389	3554.980ep ±0.700	7516.130cs ±0.787	9547.883br ±0.576
**3**	1288.293hb hc ±0.320	2043.430gc ±0.767	5459.497du ±0.496	9765.663bp ±0.534	12346.710ai ±0.830
**4**	1603.033gr ±0.338	2491.790fp ±0.707	7129.957db ±1.318	11373.147aw ±0.974	14144.367q ±0.951
**5**	1803.737gl gm ±0.558	2822.287ff ±0.935	8215.717ci ±0.863	12425.110ah ±1.096	15218.610j ±0.897
**6**	1956.170gg ±0.328	3116.957ey ±0.728	8742.110cd ±1.524	12946.800aa ±0.765	15945.863e ±0.662
**7**	2093.567ga ±0.696	3288.410et ±0.380	9029.650by ±1.934	13223.477z ±1.492	16485.853b ±1.527
**8**	2136.473fz ±0.488	3302.093es ±0.926	9161.203bv ±1.979	13405.923x ±1.846	16782.397a ±0.716

Note: Different group letters that follow the means prescribe statistically significant means. Results were calculated with *post-hoc* Duncan (P = 0.05) multiple comparisons test, within the two-way ANOVA test (P = 0.05).

Supplementary Table S9. Percentage of swelling index (idxSWL (%)) from microspheres

The results belong to the P5*pH*Time interaction factor levels

Time (h)	P5_pH1.2	P5_pH3	P5_pH5	P5_pH6.8	P5_pH7.4
1	384.983if ±0.418	445.157ic id ±0.289	784.587hr ±0.283	2618.067fl fm ±0.570	3948.940el ±0.255
2	732.370ht ±0.835	894.810hn ±0.485	2178.903fx ±0.406	5229.780dx ±0.584	7054.697dc ±0.230
3	1078.823hi ±0.776	1288.540hb hc ±0.721	3591.763eo ±0.565	7498.550ct ±0.687	9772.807bp ±0.525
4	1365.343gy gz ±0.658	1684.603gp ±0.278	4959.020dz ±0.428	9093.400bw ±1.003	11480.290au ±0.964
5	1572.137gs ±0.593	2060.910gb ±0.632	6056.623dp ±0.701	9979.607bm ±0.752	12625.623ae ±0.836
6	1729.663go ±1.223	2451.150fr ±1.549	6992.553de ±0.429	10536.827bf ±0.752	13490.070u ±0.9070
7	1794.250gm gn ±0.478	2641.250fk ±0.446	7443.593cv ±1.513	10983.810ba ±2.091	14149.643q ±1.351
8	1803.763gl gm ±0.435	2802.273fg ±0.835	7557.760cr ±2.066	11268.263ay ±1.857	14321.463p ±0.341

Note: Different group letters that follow the means prescribe statistically significant means. Results were calculated with *post-hoc* Duncan (P = 0.05) multiple comparisons test, within the two-way ANOVA test (P = 0.05).

Supplementary Table S10. Percentage of swelling index (idxSWL (%)) from microspheres
The results belong to the P6*pH*Time interaction factor levels

Time	P6_pH1.2	P6_pH3	P6_pH5	P6_pH6.8	P6_pH7.4
**1h**	379.303if ±0.202	407.220ie ±1.874	649.597hw ±0.225	2481.540fp ±0.185	3747.473em ±0.371
**2h**	678.393hv ±0.462	854.020hp ±0.673	2057.203gb gc ±0.248	4867.977ea ±0.543	6987.917de ±0.636
**3h**	928.083hm ±0.160	1281.093hc ±0.103	3396.293er ±0.272	6724.627dg ±0.702	9417.887bs ±0.605
**4h**	1174.967hf ±0.158	1677.577gp gq ±0.681	4693.370ed ±0.957	8382.453ch ±0.275	11289.077ax ±0.362
**5h**	1360.163gz ±0.335	2048.117gb gc ±0.706	5756.450ds ±1.019	9593.530bq ±0.929	12265.020ak ±0.531
**6h**	1454.693gx ±0.678	2384.230fs ±0.520	6652.060dh ±0.931	10434.770bi ±0.518	12934.150aa ±5.181
**7h**	1521.520gu ±0.215	2588.487fn ±0.376	7145.203da ±1.778	10886.183bc ±1.025	13474.850v ±1.343
**8h**	1548.223gt ±0.224	2723.563fh ±0.314	7262.257cy ±0.545	11044.960az ±1.075	13734.510r ±1.117

Note: Different group letters that follow the means prescribe statistically significant means. Results were calculated with *post-hoc* Duncan (P = 0.05) multiple comparisons test, within the two-way ANOVA test (P = 0.05).

Supplementary Table S11. Percentage of swelling index (idxSWL (%)) from microspheres

The results belong to the P7*pH*Time interaction factor levels

Time (h)	P7_pH1.2	P7_pH3	P7_pH5	P7_pH6.8	P7_pH7.4
1	308.770ig ±0.387	387.670if ±0.653	626.213hy hz ±0.284	2094.073ga ±0.737	3533.300eq ±0.256
2	596.240ia ±0.159	802.433hq ±0.403	1901.897gh ±0.285	4445.310eg ±0.675	6546.773dj ±0.543
3	853.373hp ±0.119	1205.470he ±0.343	3237.780ev ±0.455	6485.967dk ±0.392	9275.717bu ±0.586
4	1041.837hj ±0.413	1612.200gr ±0.255	4394.020eh ±0.725	7994.533cm ±1.052	11052.050az ±1.378
5	1166.590hf ±0.273	2004.283ge ±0.556	5301.637dw ±0.349	9049.467bx ±1.014	12155.257am ±0.907
6	1248.843hd ±0.440	2346.220ft ±0.236	5990.780dq ±1.082	9828.100bo ±0.744	12786.843ac ±0.192
7	1297.750hb ±0.385	2561.877fo ±0.419	6428.593dl ±1.169	10198.543bl ±0.740	13347.203y ±1.054
8	1323.147ha ±1.537	2606.533fm ±0.111	6616.847di ±0.661	10260.023bk ±1.305	13632.090t ±1.579

Note: Different group letters that follow the means prescribe statistically significant means. Results were calculated with *post-hoc* Duncan (P = 0.05) multiple comparisons test, within the two-way ANOVA test (P = 0.05).

Supplementary Table S12. Percentage of swelling index (idxSWL (%)) from microspheres

The results belong to the P8*pH*Time interaction factor levels

Time (h)	P8_pH1.2	P8_pH3	P8_pH5	P8_pH6.8	P8_pH7.4
**1**	410.690ie ±0.608	449.950ic ±0.338	802.647hq ±0.329	2720.317fh ±0.620	4079.207ek ±0.251
**2**	769.073hs ±0.307	993.303hk ±0.414	2293.713fu ±0.574	5441.483dv ±0.913	7382.173cx ±0.739
**3**	1095.507hh ±0.260	1498.203gv ±0.833	3753.277em ±0.175	7640.420cq ±0.848	9914.377bn ±0.651
**4**	1376.493gy ±0.368	1892.430gh ±0.245	5010.570dy ±0.896	9325.203bt ±1.426	11656.660as ±0.436
**5**	1612.373gr ±0.539	2279.257fv fw ±0.691	6082.457do ±0.564	10318.707bj ±1.146	12856.863ab ±47.953
**6**	1796.710gm gn ±0.559	2591.740fn ±4.789	7013.023dd ±1.283	10949.133bb ±1.019	13673.733s ±0.344
**7**	1894.040gh ±0.400	2803.477fg ±0.713	7655.807cp ±1.248	11393.470av ±0.956	14354.557o ±0.445
**8**	1972.503gf ±0.478	2975.593fc ±0.721	8091.447ck ±0.871	11587.580at ±1.675	14579.537m ±0.847

Note: Different group letters that follow the means prescribe statistically significant means. Results were calculated with *post-hoc* Duncan (P = 0.05) multiple comparisons test, within the two-way ANOVA test (P = 0.05).

Supplementary Table S13. Percentage of QUE released (mQrel (%)) from microspheres

The results belong to the Sample*Time interaction factor levels

Time (h)	P1	P2	P3	P5	P6	P7	QUE
**0.25**	0.150bh ±0.008	0.290bh ±0.014	0.387bh ±0.013	0.120bh ±0.008	0.117bh ±0.013	0.130bh ±0.008	1.820bg ±0.269
**0.5**	11.187bc ±0.017	14.473bb ±0.005	15.067ba ±0.033	6.963bf ±0.029	7.480be ±0.008	8.187bd ±0.013	24.467av ±0.314
**0.75**	22.460aw ±0.028	26.347at ±0.021	28.780ar ±0.025	16.377az ±0.013	17.853ay ±0.029	18.937ax ±0.033	41.477am ±0.338
**1**	35.143ap ±0.009	38.053ao ±0.017	42.090al ±0.008	25.177au ±0.033	28.167as ±0.013	30.353aq ±0.029	55.440ae ±0.385
**2**	59.517ai ±0.033	63.357ag ±0.042	67.810af ±0.008	44.717ap ±0.013	49.573an ±0.029	56.087ak ±0.013	76.073y ±0.707
**3**	77.850ac ±0.022	81.843aa ±0.021	85.193x ±0.021	64.687aj ±0.013	69.277ah ±0.042	74.643ad ±0.029	89.107t ±0.522
**4**	85.103w ±0.034	87.917u ±0.017	90.227r ±0.017	71.547ae ±0.033	76.270ab ±0.008	80.347y ±0.042	96.217n ±0.682
**5**	90.337rs ±0.013	92.417o ±0.039	94.917l ±0.033	77.540z ±0.008	80.783w ±0.029	85.140u ±0.008	97.320h ±0.650
**6**	92.830l ±0.008	94.347j ±0.009	96.483g ±0.021	81.777v ±0.013	84.907t ±0.013	88.443q ±0.029	97.337b ±0.725
**8**	96.143g ±0.009	96.140e ±0.008	97.733c ±0.021	84.597s ±0.013	87.377p ±0.013	90.913l ±0.029	97.347a ±0.723
**12**	47.273e ±0.041	51.097d ±0.031	54.307b ±0.013	35.120o ±0.008	38.850lm ±0.008	43.377i ±0.033	66.847a ±0.473
**24**	69.647b ±0.009	74.387b ±0.009	78.017a ±0.039	55.133m ±0.029	60.357k ±0.033	66.450f ±0.008	84.050a ±0.611

Note: Different letters that follow the means prescribe statistically significant means. Results were calculated with *post-hoc* Duncan (P = 0.05) multiple comparisons test, within the two-way ANOVA test (P = 0.05).

Supplementary Table S14. Percentage of QUE released (mQUErel (%)) from microsphere

The results belong to the Time interaction factor levels

Time (h)	mQUErel_t (%)
**0.25**	0.430l ±0.584
**0.5**	12.546k ±5.732
**0.75**	24.604j ±8.065
**1**	36.346i ±9.484
**2**	48.124h ±9.829
**3**	59.590g ±9.912
**4**	69.720f ±9.316
**5**	77.514e ±8.046
**6**	83.947d ±7.867
**8**	88.350c ±6.855
**12**	90.875b ±5.528
**24**	92.893a ±4.900

Note: Different letters that follow the means prescribe statistically significant means. Results were calculated with *post-hoc* Duncan (P = 0.05) multiple comparisons test, within the two-way ANOVA test (P = 0.05).

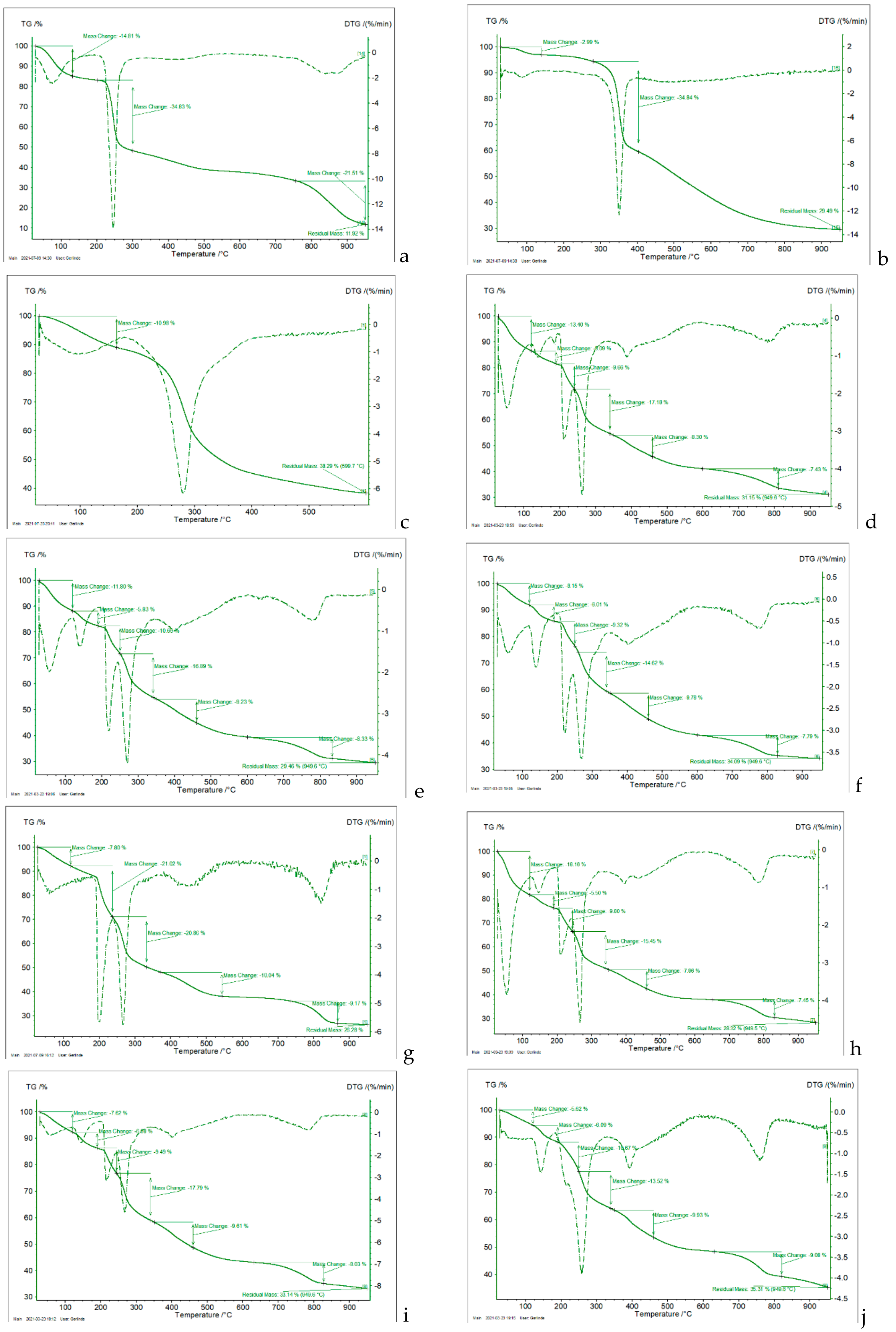
TG /%
DTG /(%/min)
Temperature /°C
a
Residual Mass: 11.92 %
b
Residual Mass: 29.49 %
c
Residual Mass: 38.29 % (599.7 °C)
d
Residual Mass: 31.15 % (949.6 °C)
e
Residual Mass: 29.46 % (949.6 °C)
f
Residual Mass: 34.09 % (949.6 °C)
g
Residual Mass: 28.28 %
h
i
Residual Mass: 33.14 % (949.6 °C)
j

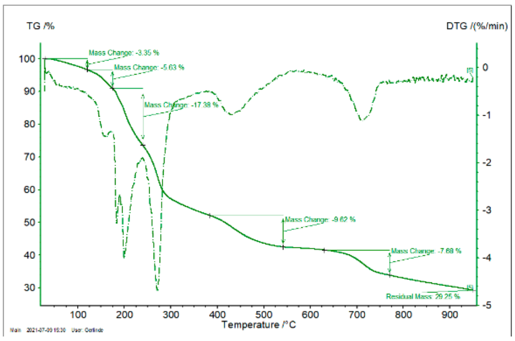

k

Supplementary Figure S2. TG and DTG for: a - Na-Alg, b – QUE, c – Ch, d – P1, e – P2, f – P3, g – P4, h – P5, i – P6, j – P7, k – P8.

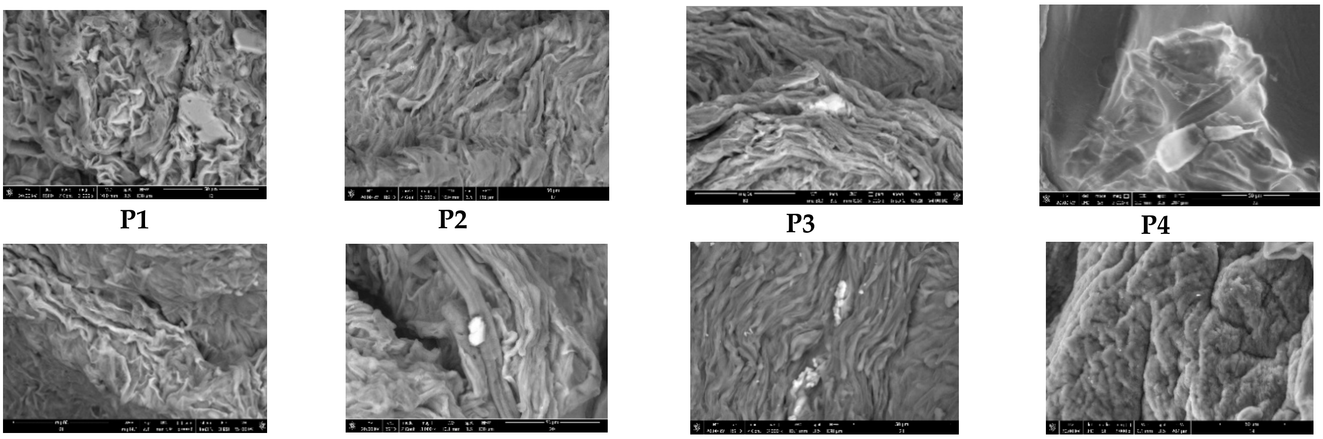

P1 **P2** **P3** **P4**

P5 **P6** **P7** **P8**

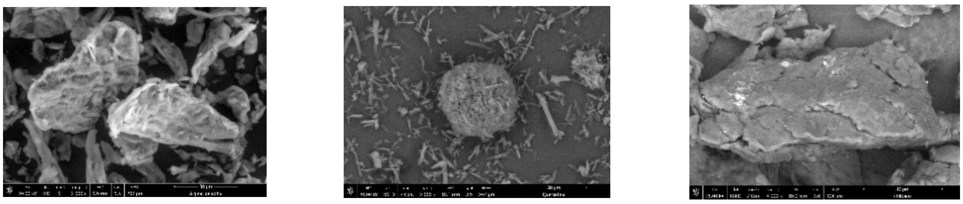

Sodium alginate **Quercetin** **Chitosan**

Supplementary Figure S3. SEM analysis of quercetin micro-spheres.

Supplementary Table S15. Results of the non-linear regression of the idxSWL (%) time series for sample P1 and all pH factor levels

P1	pH 1.2	pH 3	pH 5	pH 6.8	pH 7.4
**Best-fit values**					
**Ymax**	2504	4322	14554	13546	17490
**K**	0.2281	0.1872	0.1332	0.3863	0.3629
**HalfLife**	3.039	3.704	5.203	1.794	1.910
**Std. Error**					
**Ymax**	55.18	113.3	1342	201.3	185.0
**K**	0.009860	0.008634	0.01854	0.01598	0.01022
**95% Confidence Intervals**					
**Ymax**	2390 to 2619	4087 to 4557	11770 to 17337	13129 to 13963	17106 to 17873
**K**	0.2076 to 0.2485	0.1692 to 0.2051	0.09477 to 0.1717	0.3532 to 0.4195	0.3417 to 0.3841
**HalfLife**	2.789 to 3.338	3.380 to 4.096	4.038 to 7.314	1.652 to 1.963	1.804 to 2.028
**Goodness of Fit**					
**Degrees of Freedom**	22	22	22	22	22
**R square**	0.9940	0.9952	0.9792	0.9873	0.9944
**Adjusted R square**	0.9937	0.9950	0.9783	0.9867	0.9941

Supplementary Table S16. Results of the non-linear regression of the idxSWL (%) time series for sample P2 and all pH factor levels

P2	pH 1.2	pH 3	pH 5	pH 6.8	pH 7.4
**Best-fit values**					
**Ymax**	2583	4036	13517	13813	17603
**K**	0.2036	0.2017	0.1395	0.3239	0.3136
**HalfLife**	3.405	3.437	4.969	2.140	2.211
**Std. Error**					
**Ymax**	60.68	90.59	1318	272.3	200.0
**K**	0.008796	0.008283	0.02091	0.01569	0.008563
**95% Confidence Intervals**					
**Ymax**	2457 to 2709	3848 to 4224	10784 to 16251	13248 to 14378	17188 to 18017
**K**	0.1853 to 0.2218	0.1845 to 0.2188	0.09614 to 0.1829	0.2913 to 0.3564	0.2958 to 0.3313
**HalfLife**	3.125 to 3.740	3.167 to 3.757	3.791 to 7.210	1.945 to 2.379	2.092 to 2.343
**Goodness of Fit**					
**Degrees of Freedom**	22	22	22	22	22
**R square**	0.9951	0.9957	0.9744	0.9867	0.9957
**Adjusted R square**	0.9949	0.9955	0.9733	0.9861	0.9955

Supplementary Table S17. Results of the non-linear regression of the idxSWL (%) time series for sample P3 and all pH factor levels

P3	pH 1.2	pH 3	pH 5	pH 6.8	pH 7.4
**Best-fit values**					
**Ymax**	2655	3819	13490	13406	16322
**K**	0.1886	0.2036	0.1261	0.2977	0.3300
**HalfLife**	3.675	3.404	5.495	2.328	2.100
**Std. Error**					
**Ymax**	74.87	63.60	1652	152.5	216.8
**K**	0.009393	0.006237	0.02283	0.007861	0.01092
**95% Confidence Intervals**					
**Ymax**	2499 to 2810	3687 to 3951	10064 to 16917	13090 to 13723	15873 to 16772
**K**	0.1691 to 0.2081	0.1907 to 0.2165	0.07880 to 0.1735	0.2814 to 0.3140	0.3074 to 0.3527
**HalfLife**	3.331 to 4.099	3.201 to 3.635	3.995 to 8.796	2.207 to 2.463	1.966 to 2.255
**Goodness of Fit**					
**Degrees of Freedom**	22	22	22	22	22
**R square**	0.9943	0.9975	0.9698	0.9964	0.9933
**Adjusted R square**	0.9941	0.9974	0.9684	0.9962	0.9930

Supplementary Table S18. Results of the non-linear regression of the idxSWL (%) time series for sample P4 and all pH factor levels

P4	pH 1.2	pH 3	pH 5	pH 6.8	pH 7.4
**Best-fit values**					
**Ymax**	2647	4070	12183	14486	17862
**K**	0.2204	0.2297	0.1978	0.3644	0.3750
**HalfLife**	3.146	3.018	3.504	1.902	1.848
**Std. Error**					
**Ymax**	60.33	100.1	853.0	199.4	207.8
**K**	0.009658	0.01112	0.02509	0.01339	0.01190
**95% Confidence Intervals**					
**Ymax**	2521 to 2772	3862 to 4277	10413 to 13952	14072 to 14899	17431 to 18293
**K**	0.2003 to 0.2404	0.2066 to 0.2527	0.1458 to 0.2499	0.3366 to 0.3922	0.3504 to 0.3997
**HalfLife**	2.883 to 3.460	2.743 to 3.355	2.774 to 4.754	1.767 to 2.059	1.734 to 1.978
**Goodness of Fit**					
**Degrees of Freedom**	22	22	22	22	22
**R square**	0.9942	0.9925	0.9657	0.9907	0.9926
**Adjusted R square**	0.9939	0.9922	0.9641	0.9902	0.9923

Supplementary Table S19. Results of the non-linear regression of the idxSWL (%) time series for sample P5 and all pH factor levels

P5	pH 1.2	pH 3	pH 5	pH 6.8	pH 7.4
**Best-fit values**					
**Ymax**	2304	5592	15307	12887	15957
**K**	0.2140	0.09050	0.09334	0.2809	0.3068
**HalfLife**	3.239	7.659	7.426	2.467	2.259
**Std. Error**					
**Ymax**	81.97	378.7	2502	281.7	160.0
**K**	0.01440	0.008136	0.02043	0.01372	0.007288
**95% Confidence Intervals**					
**Ymax**	2134 to 2474	4806 to 6377	10119 to 20495	12303 to 13471	15625 to 16289
**K**	0.1842 to 0.2439	0.07362 to 0.1074	0.05098 to 0.1357	0.2525 to 0.3094	0.2917 to 0.3219
**HalfLife**	2.842 to 3.764	6.456 to 9.415	5.108 to 13.60	2.240 to 2.745	2.153 to 2.376
**Goodness of Fit**					
**Degrees of Freedom**	22	22	22	22	22
**R square**	0.9874	0.9955	0.9749	0.9894	0.9969
**Adjusted R square**	0.9869	0.9953	0.9738	0.9889	0.9968

Supplementary Table S20. Results of the non-linear regression of the idxSWL (%) time series for sample P6 and all pH factor levels

P6	pH 1.2	pH 3	pH 5	pH 6.8	pH 7.4
**Best-fit values**					
**Ymax**	1874	5275	15774	13464	15122
**K**	0.2387	0.09519	0.08428	0.2353	0.3211
**HalfLife**	2.904	7.281	8.224	2.946	2.158
**Std. Error**					
**Ymax**	40.97	381.6	2977	304.3	175.4
**K**	0.01051	0.009273	0.02072	0.01063	0.009104
**95% Confidence Intervals**					
**Ymax**	1789 to 1959	4483 to 6066	9599 to 21950	12833 to 14095	14758 to 15485
**K**	0.2169 to 0.2605	0.07596 to 0.1144	0.04131 to 0.1273	0.2133 to 0.2573	0.3023 to 0.3400
**HalfLife**	2.661 to 3.196	6.058 to 9.125	5.447 to 16.78	2.694 to 3.250	2.039 to 2.293
**Goodness of Fit**					
**Degrees of Freedom**	22	22	22	22	22
**R square**	0.9931	0.9943	0.9743	0.9931	0.9953
**Adjusted R square**	0.9928	0.9940	0.9732	0.9928	0.9951

Supplementary Table S21. **Results of the non-linear regression of the idxSWL (%) time series for sample P7 and all pH factor levels**

P7	pH 1.2	pH 3	pH 5	pH 6.8	pH 7.4
**Best-fit values**					
**Ymax**	1552	5373	12765	12579	15279
**K**	0.2622	0.08933	0.09905	0.2378	0.3009
**HalfLife**	2.644	7.759	6.998	2.914	2.303
**Std. Error**					
**Ymax**	33.22	601.3	1932	405.2	240.5
**K**	0.01199	0.01322	0.02043	0.01541	0.01107
**95% Confidence Intervals**					
**Ymax**	1483 to 1621	4126 to 6620	8758 to 16773	11738 to 13419	14781 to 15778
**K**	0.2373 to 0.2870	0.06191 to 0.1168	0.05669 to 0.1414	0.2059 to 0.2698	0.2780 to 0.3239
**HalfLife**	2.415 to 2.921	5.937 to 11.20	4.901 to 12.23	2.569 to 3.367	2.140 to 2.494
**Goodness of Fit**					
**Degrees of Freedom**	22	22	22	22	22
**R square**	0.9916	0.9885	0.9752	0.9862	0.9930
**Adjusted R square**	0.9912	0.9880	0.9740	0.9856	0.9927

Supplementary Table S22. Results of the non-linear regression of the idxSWL (%) time series for sample P8 and all pH factor levels

P8	pH 1.2	pH 3	pH 5	pH 6.8	pH 7.4
**Best-fit values**					
**Ymax**	2622	4938	18164	13352	16089
**K**	0.1836	0.1197	0.07759	0.2786	0.3142
**HalfLife**	3.775	5.791	8.933	2.488	2.206
**Std. Error**					
**Ymax**	61.55	239.8	2695	279.0	117.4
**K**	0.007509	0.008425	0.01469	0.01293	0.005518
**95% Confidence Intervals**					
**Ymax**	2494 to 2750	4441 to 5435	12575 to 23752	12773 to 13930	15845 to 16332
**K**	0.1680 to 0.1992	0.1022 to 0.1372	0.04713 to 0.1081	0.2518 to 0.3054	0.3028 to 0.3257
**HalfLife**	3.480 to 4.125	5.053 to 6.781	6.415 to 14.71	2.270 to 2.753	2.128 to 2.289
**Goodness of Fit**					
**Degrees of Freedom**	22	22	22	22	22
**R square**	0.9963	0.9953	0.9865	0.9905	0.9982
**Adjusted R square**	0.9962	0.9951	0.9859	0.9900	0.9981

Supplementary Table S23. Results of the non-linear regression of the QUE *in vitro* release time series for samples P1-P7 and QUE

mQUErel (%)	P1	P2	P3	P5	P6	P7	P1
**Best-fit values**							
**Ymax**	95.69	95.91	97.37	85.88	88.30	91.50	97.85
**K**	0.3412	0.3877	0.4217	0.2698	0.2967	0.3255	0.6390
**HalfLife**	2.032	1.788	1.644	2.569	2.336	2.130	1.085
**Std. Error**							
**Ymax**	1.342	1.276	1.374	1.244	1.275	1.229	1.392
**K**	0.01356	0.01550	0.01857	0.009974	0.01140	0.01213	0.03356
**95% Confidence Intervals**							
**Ymax**	92.96 to 98.42	93.31 to 98.51	94.57 to 100.2	83.35 to 88.41	85.71 to 90.89	89.00 to 94.00	95.02 to 100.7
**K**	0.3136 to 0.3687	0.3562 to 0.4192	0.3839 to 0.4595	0.2495 to 0.2901	0.2735 to 0.3199	0.3008 to 0.3501	0.5708 to 0.7073
**HalfLife**	1.880 to 2.210	1.654 to 1.946	1.509 to 1.805	2.390 to 2.778	2.167 to 2.534	1.980 to 2.304	0.9800 to 1.214
**Goodness of Fit**							
**Degrees of Freedom**	34	34	34	34	34	34	34
**R square**	0.9886	0.9880	0.9852	0.9906	0.9897	0.9903	0.9754
**Adjusted R square**	0.9882	0.9877	0.9848	0.9903	0.9894	0.9901	0.9747

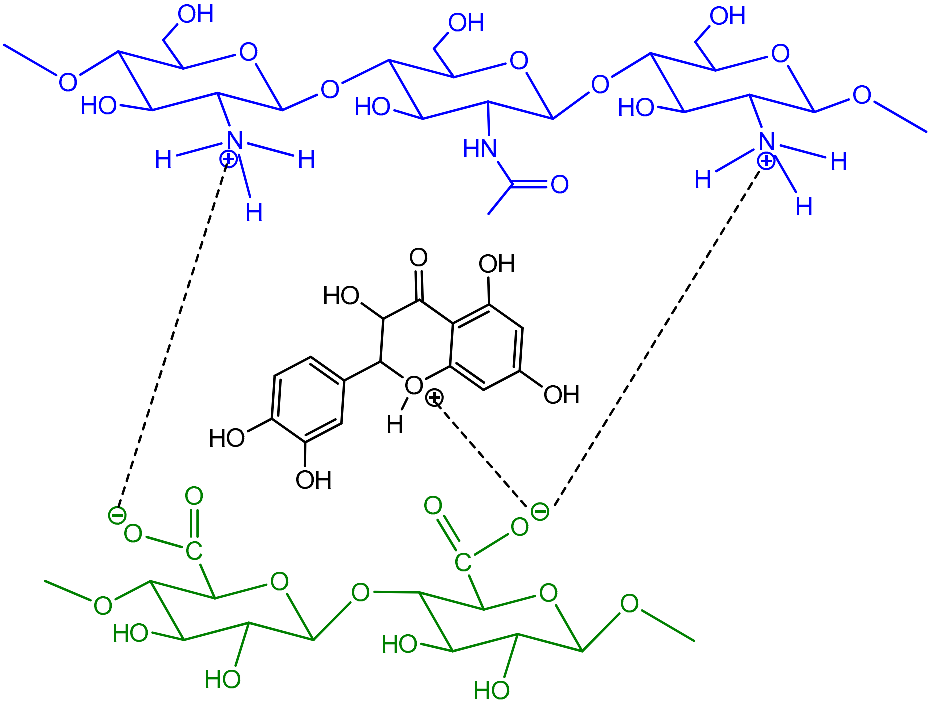

Supplementary Figure S4 – Interactions established during microsphere formation and drug loading.

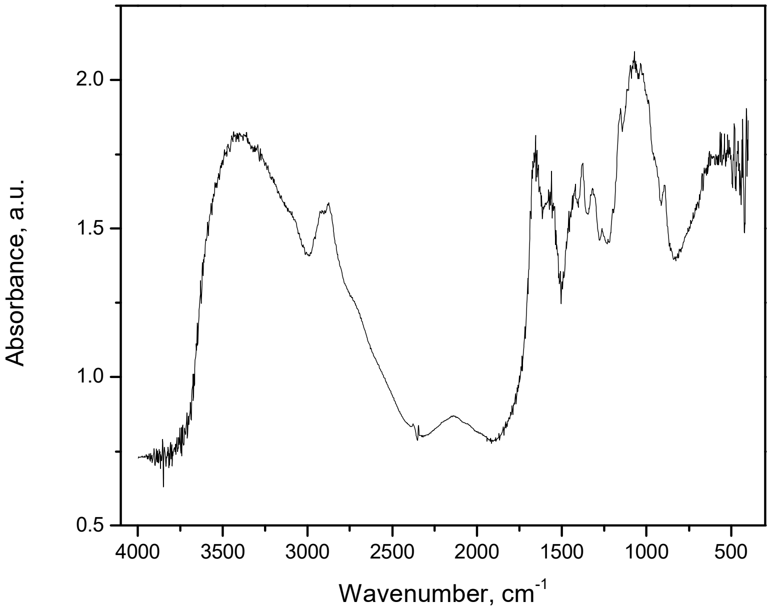

Supplementary Figure S5 – Ch FT-IR spectra

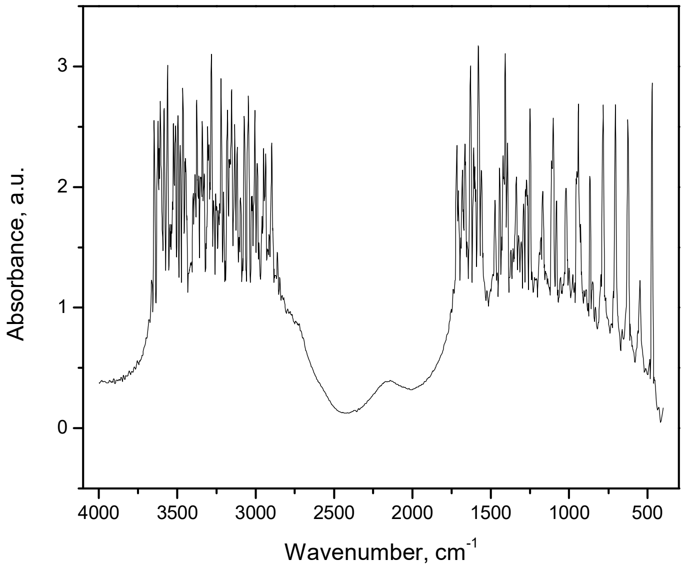

Supplementary Figure S6 – Na-Alg FT-IR spectra

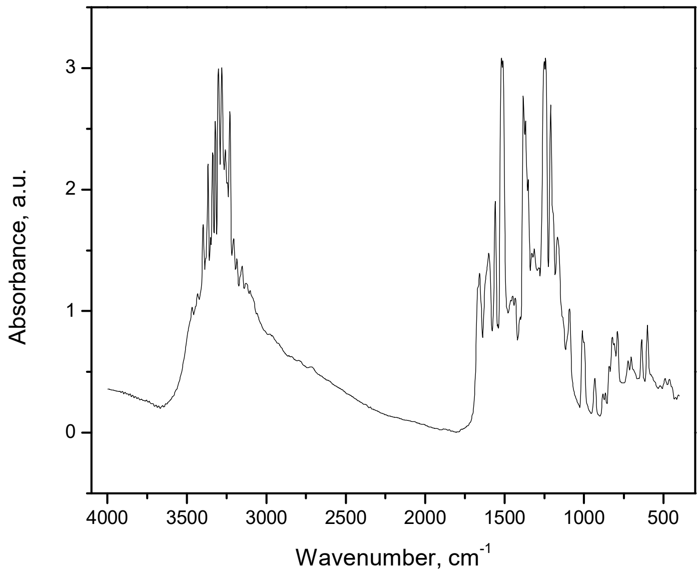

Supplementary Figure S7 – QUE FT-IR spectra

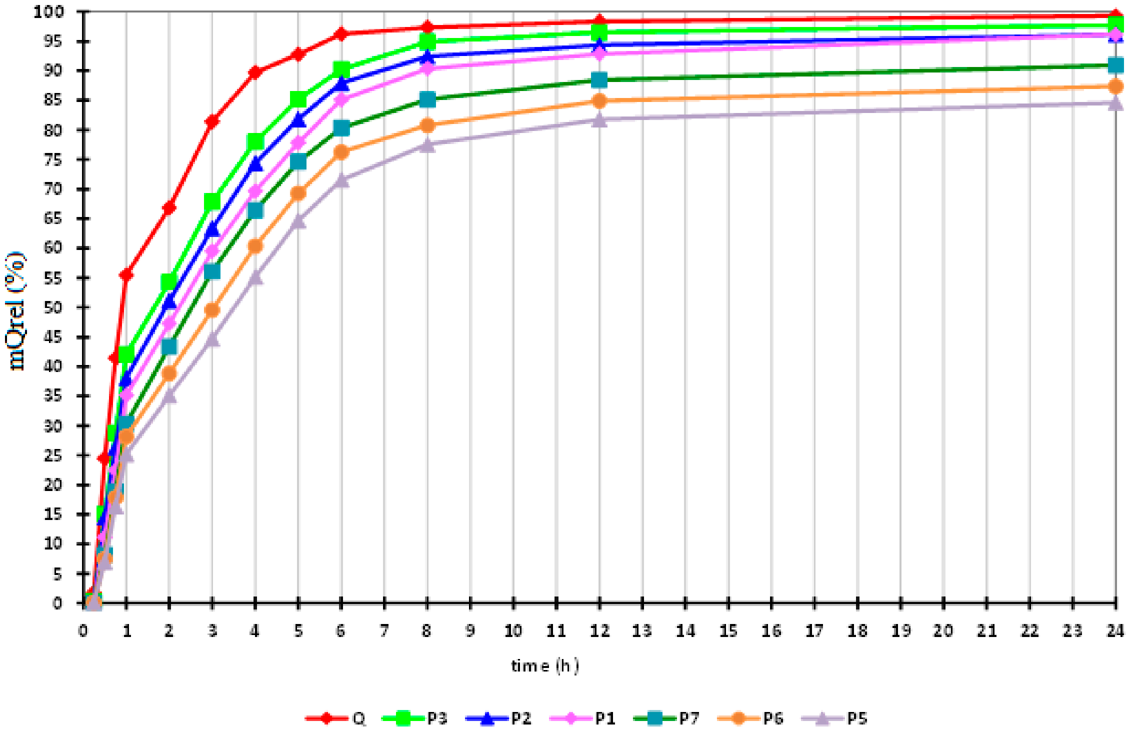

Supplementary Figure S8 - Interval plots of the microspheres' percentage of Q released (mQrel (%)) from microspheres for Sample*Time interaction factor levels
